# Different cortical connectivities in human females and males relate to differences in strength and body composition, reward and emotional systems, and memory

**DOI:** 10.1007/s00429-023-02720-0

**Published:** 2023-10-20

**Authors:** Ruohan Zhang, Edmund T. Rolls, Wei Cheng, Jianfeng Feng

**Affiliations:** 1 Department of Computer Science, University of Warwick, Coventry, CV4 7AL UK; 2 Institute of Science and Technology for Brain Inspired Intelligence, Fudan University, Shanghai, 200403 China; 3 Oxford Centre for Computational Neuroscience, Oxford, UK

**Keywords:** Sex differences in the brain, Functional connectivity, Ventromedial prefrontal cortex, Liking for sweet foods, Somatosensory and motor cortex

## Abstract

Sex differences in human brain structure and function are important, partly because they are likely to be relevant to the male–female differences in behavior and in mental health. To analyse sex differences in cortical function, functional connectivity was measured in 36,531 participants (53% female) in the UK Biobank (mean age 69) using the Human Connectome Project multimodal parcellation atlas with 360 well-specified cortical regions. Most of the functional connectivities were lower in females (Bonferroni corrected), with the mean Cohen’s d =  − 0.18. Removing these as covariates reduced the difference of functional connectivities for females—males from *d* =  − 0.18 to − 0.06. The lower functional connectivities in females were especially of somatosensory/premotor regions including the insula, opercular cortex, paracentral lobule and mid-cingulate cortex, and were correlated with lower maximum workload (*r* = 0.17), and with higher whole body fat mass (*r* =  − 0.17). But some functional connectivities were higher in females, involving especially the ventromedial prefrontal cortex and posterior cingulate cortex, and these were correlated with higher liking for some rewards such as sweet foods, higher happiness/subjective well-being, and with better memory-related functions. The main findings were replicated in 1000 individuals (532 females, mean age 29) from the Human Connectome Project. This investigation shows the cortical systems with different functional connectivity between females and males, and also provides for the first time a foundation for understanding the implications for behavior of these differences between females and males.

**Supplementary Information:**

The online version contains supplementary material available at 10.1007/s00429-023-02720-0.

## Introduction

Sex differences in human brain structure and function are important, partly because they are likely to be relevant to the male–female differences in autism spectrum disorder (Werling and Geschwind 2013; Baio et al. 2018; Henry et al. 2018), Alzheimer’s disease (Vina and Lloret 2010; Mazure and Swendsen 2016), major depressive disorder (Rutter et al. 2003; Gobinath et al. 2017; Zhang et al. 2020), tobacco smoking (McCarthy et al. 2019), personality (Li et al. 2020), and internet gaming (Zeng et al. 2021). Sex differences have been observed in brain structure. Males had higher cortical and subcortical volumes, and cortical surface areas (Ruigrok et al. 2014; Ritchie et al. 2018), with the different regions including the medial and lateral prefrontal, orbitofrontal, superior temporal, and lateral parietal cortices and insula (Ruigrok et al. 2014; Liu et al. 2020). Functional magnetic resonance imaging (fMRI) studies found sex differences in functional connectivity (FC), measured by the correlation between the BOLD signal in different brain regions and reflecting brain connectivity and function (Rolls 2023c), with for example higher FCs in males than females, involving the frontal, parietal, and temporal lobes (Zhang et al. 2016). Males were found to have higher FCs related to the sensorimotor network, while females had higher FCs related to the default mode network (Ritchie et al. 2018). Most of these studies used resting-state fMRI data with fewer than 1000 healthy participants, and the automated anatomical labelling (AAL) atlas for fMRI data parcellation (Rolls et al. 2015). We note that large sample sizes are needed to ensure robust results in association studies (Jiao et al. 2022; Marek et al. 2022).

With this background, one of the aims of the present investigation is to measure functional connectivity in females and males with a high resolution cortical parcellation in which the different regions have interpretable functions (Rolls 2023c), the Human Connectome Project Multimodal Parcellation, which by using structural cortical measures (cortical myelin and thickness), functional connectivity, and task-related fMRI has been able to define 360 cortical regions each with potentially different connectivity and functions (Glasser et al. 2016a; Huang et al. 2022). (The functions of many of the regions defined in this atlas are interpretable in that many correspond to regions defined anatomically and physiologically in macaques and now in humans, and the great body of neuroscience research on these well-defined regions can be brought to bear to understand their functions Glasser et al. 2016a; Kandel et al. 2021; Rolls 2023c).) A key advantage of this atlas is that the effective connectivity, functional connectivity, and anatomical connectivity measured with diffusion tractography for most of the cortical regions is now available, and this helps with the interpretation of the findings when this HCP-MMP atlas is utilized (Rolls et al. 2022a, 2022b; Rolls et al. 2023a, b; Rolls et al. 2023c, d, e; Rolls et al. 2023a, b). The functional connectivity is a useful measure that may reflect the interactive coupling between cortical regions, and may reflect how much the cortical regions influence each other or are influenced by common input (Rolls 2023c). A second aim was to use a sufficiently large dataset to obtain reliable results with this atlas with functional connectivities measured between all 360 cortical regions, and this aim was realised by the use of resting state fMRI data from a very large sample of 36,531 participants from the UK Biobank (Rusk 2018). A third aim was to investigate what possible behavioural measures might be associated with the sex differences in functional connectivities. This is a key new aim of the research described here, and is important so that the differences in functional connectivity between females and males can be interpreted in terms of the behavioural differences between females and males. A fourth aim was to perform a sex prediction analysis using the top significant functional connectivity differences, as a validation of the sex differences found. A fifth aim was to test whether the main findings could be replicated using the resting-state fMRI data of 1000 participants from the Human Connectome Project (Glasser et al. 2016b) in which the age is lower than in the UK Biobank participants, in order to discover whether the sex differences in functional connectivity between females and males are consistent in these different age groups.

The hypotheses tested were that sex differences could be found for functional connectivities that are different for different cortical regions, and that these differences could be related to differences in behavior and body measures between females and males. We further tested the hypotheses that any differences found are related to measurable differences between females and males, including the greater physical strength and lower body fat of males, and the greater liking of sweet foods of females and their wellbeing (across the population), which as far as we know have not been analysed in previous research.

The key aims of the study thus included assessment of differences in cortical functional connectivity between human females and males using the Human Connectome Project Multimodal Parcellation atlas with its clear identification of 360 different cortical regions; use of a very large sample size to enable adequate statistical assessment of the differences found; and analysis of any behavioral associations of the differences found, to help interpret the differences in functional connectivity.

## Methods

### Study population

The dataset used for this study was obtained from the UK Biobank (the September 2019 public data release), which includes a wide range of phenotypic information and biological samples of ∼500,000 participants who attended 1 of 22 assessment centers across the United Kingdom from 2006 to 2010 (Miller et al. 2016). The UK Biobank received ethical approval from the North West Multi-Centre Research Ethics Committee in the UK (REC reference 11/NW/0382). The present analyses were conducted under UK Biobank application number 19542. Written informed consent was obtained from each participant according to the Declaration of Helsinki. The UK Biobank collected multi-modal neuroimaging data from ~ 40,000 participants, using identical MRI scanners at four dedicated sites, to maximise data homogeneity. In the current study, we used brain imaging data collected from the three imaging centers in which most of the brain imaging was performed, and this simplifies the use of imaging site as a covariate. After quality controls on the resting-state functional brain imaging data and removing some participants without a complete measurement of demographic characteristics (including age, body mass index or BMI, education qualifications, Townsend social deprivation index, alcohol drinker status, and smoking status), 36,531 participants of the UKB sample remained in the present neuroimaging analyses. The demographic characteristics and brain imaging confound-regressor variables (including head motion and brain imaging site information) of participants are summarized in Table 1. The participants were not selected on the basis of their health, but in practice, there is evidence of “healthy volunteer bias” in the UK Biobank, i.e., participants are less likely to smoke, be obese, and consume alcohol daily than the general UK adults (Fry et al. 2017).

**Table 1:** Demographic characteristics of the 36,531 UK Biobank participants

Characteristics	No. (%)
Age, mean (SD), years	68.74 (7.44)
Female	19,396 (53.09%)
BMI, mean (SD), kg/m^2^	26.55 (4.21)
Townsend deprivation index, mean (SD), points	− 1.90 (2.71)
Education Qualifications	
College or University degree	16,999 (46.53%)
A levels/AS levels or equivalent	4812 (13.17%)
O levels/GCSEs or equivalent	7047 (19.29%)
CSEs or equivalent	1497 (4.10%)
NVQ or HND or HNC or equivalent	1970 (5.39%)
Other professional qualifications e.g.: nursing, teaching	1814 (4.97%)
None of the above	2392 (6.55%)
Alcohol drinker status	
Never	907 (2.48%)
Previous	773 (2.12%)
Current	34,843 (95.38%)
Smoking status	
Never	22,306 (61.06%)
Previous	11,962 (32.74%)
Current	2263 (6.19%)
Head motion, mean (SD), mm	0.12 (0.06)
Site information	
Site 1	22,356 (61.20%)
Site 2	4861 (13.31%)
Site 3	9314 (25.49%)

The data from the Human Connectome Project were obtained by the WU-Minn HCP Consortium which obtained full informed consent from all participants, and research procedures and ethical guidelines were followed in accordance with the Institutional Review Boards (IRB), with details at the HCP website (http://www.humanconnectome.org/).

### Neuroimaging

The multi-modal imaging was collected using a standard Siemens Skyra 3 T running VD13A SP4, with a standard Siemens 32-channel RF receive head coil. The resting-state fMRI data used in this study were obtained and pre-processed by the UK Biobank. The resting-state fMRI data are of 6-min duration (490 images, TR = 0.735 s), with a spatial resolution of 2.4 mm isotropic, and were acquired with 8 × multislice acceleration. The details of the image acquisition are provided on the UK Biobank website in the form of a protocol (http://biobank.ctsu.ox.ac.uk/crystal/refer.cgi?id=2367). The UK Biobank conducted all the quality checking and data pre-processing procedures and the details of the pre-processing are available on the UK Biobank website (http://biobank.ctsu.ox.ac.uk/crystal/refer.cgi?id=1977) and elsewhere (Miller et al. 2016; Alfaro-Almagro et al. 2018). Briefly, data pre-processing was carried out using FSL (FMRIB Software Library) (Jenkinson et al. 2012). All the data pre-processing procedures were performed by the UK Biobank team as described in (Miller et al. 2016), with details in the Supplementary Material.

### Behaviour assessment

The UK Biobank provides a wide range of behavioral assessments. In the current study, we further tested the hypotheses that any differences found are related to measurable differences between females and males. The behavioral assessments tested in our study included physical measures, liking for sweet food, happiness/well-being, and the cognitive task Tower rearranging.

### Physical measures

We utilized the maximum workload during fitness test (Field 6032) and whole body fat mass (Field23100) as two representative physical assessments (https://biobank.ctsu.ox.ac.uk/showcase/label.cgi?id=100012). The maximum workload during fitness test involves three distinct phases within the Bike Test protocol. The unit of this measure is watts. First, there is the Pre-Test phase, where a resting electrocardiograph (ECG) is recorded over a period of time. Following that, the Activity phase ensues, during which the participant engages in cycling for a designated duration with progressively increasing workload. Lastly, the Recovery phase occurs once the participant has ceased pedalling, and an ECG is recorded. The maximum workload during fitness test is a measure derived from the Activity phase, indicating the peak level of cycling effort achieved by the participant during this portion of the test. The whole body fat mass is one of the whole-body bio-impedance measures using the Tanita BC418MA body composition analyser.

### Liking for sweet food

This measure reflected the participants' preference for sweet food, which was measured as the mean of the preference scores for several sweet foods from category 1039, including apple juice (Field 20,602), biscuits (Field 20,615), cake (Field 20,631), icing (Field 20,632), honey (Field 20,677), chocolate (Field 20,691), orange juice (Field 20,694), and liking for sweet foods (Field 20,732).

### Happiness/well-being

The quantitative measure of this mental health symptom was obtained by calculating an average score of the items used to assess Happiness and subjective well-being (Category 147). Specifically, the scores of the three items in this category (including general happiness, general happiness with own health, and belief that own life is meaningful) were firstly adjusted to the same direction, with higher value indicating better well-being. Next, each item was normalized into a range of (0,1), and then the three items were averaged to generate an overall measure for happiness/well-being.

### Cognitive task (Tower rearranging)

This is a cognitive test which probably has a memory requirement. We used the measure of the number of puzzles correct (Field 21,004) in the task to represent the memory level of participants.

### Construction of the whole-brain functional connectivity network

After pre-processing, the cortical grey matter was parcellated into the 360 cortical regions in the HCPex atlas (Huang et al. 2022), as listed in Supplementary Table S1. The HCPex atlas is a modified and extended version of the surface-based Human Connectome Project-MultiModal Parcellation atlas of human cortical areas (HCP-MMP v1.0) (Glasser et al. 2016a), which includes 360 cortical areas including the hippocampal system to be used with the volumetric data available from the UK Biobank. For each participant, the resting state time series with 490 time points were extracted by determining the mean of the signals of all voxels within each cortical region in the atlas. Then, Pearson cross-correlations between all pairs of regional blood oxygen level-dependent signals were calculated, followed by z transformation to improve normality (Finn et al. 2015; Rosenberg et al. 2016). Then, the functional connectivity network of the left hemisphere ($180\times 180$ regions with $\frac{180\times (180-1)}{2}=16110$ links) was constructed. An analysis for the right hemisphere was also performed, for comparison. The analyses were performed separately for the left and right hemispheres in case there were differences, but the analysis for the 360 cortical regions is provided in Fig. S7 to allow assessment of differences in the interhemispheric connectivities.

### t-test on functional connectivity between females and males

To find which functional connectivities differ between females and males, two-sample t-tests were performed to compare each link in the $180\times 180$ HCPex functional connectivity matrices for the two groups, using Bonferroni correction for multiple comparisons, with age, BMI, education qualifications, smoking status, drinker status, Townsend deprivation index, head motion and site information regressed out as covariates of no interest. Further, we also found only minor differences between the males and females in these covariates, with for example little difference in head motion (females = 0.12, males = 0.13) and age (females = 68.1, males = 69.4). The effect size with Cohen’s d (the number of standard deviations between the means of the two groups) was also measured. Thereby, a $180\times 180$ *t*-value matrix and a $180\times 180$ Cohen's d-value matrix for the difference of the functional connectivities between females and males were obtained. Because 98.4% of the functional connectivity links were positive (15,850/16110), we did not attempt to analyze the positive and negative functional connectivity links separately.

### Prediction of sex from the functional connectivity using a Support Vector Machine

To help to establish the strength of the results found, we performed an analysis to predict how well the sex of each participant could be predicted from the functional connectivity. The prediction analysis for sex based on functional connectivities was performed using a linear support vector machine (SVM) approach (Meier et al. 2012; Knorr et al. 2020; Shao et al. 2020). The analyses were conducted using a MATLAB toolbox function fitclinear (Statistics and Machine Learning Toolbox Release 2021b, The MathWorks, Inc., Natick, Massachusetts, United States). Specifically, the data from 36,531 participants (19,396 females and 17,135 males) were first split randomly into a training set (80%) and a test set (20%). The sex ratio was maintained the same between the two sets. Then, the top 10% of the links with different functional connectivities between females and males identified in the training set were selected as features for sex prediction from the test set (to avoid double dipping). Additionally, a five-fold cross-validation method was used in the model training to find the optimal parameters for the SVM. This cross-validation approach was conducted by dividing the training set into five folds, using four of them for training and the remaining one for testing in a systematic and iterative manner, which could obtain a robust set of parameters for the SVM classifier, while minimizing the risk of overfitting. Moreover, the prediction accuracy was measured via the Area Under the Curve (AUC), which is defined as the area under the ROC curve. The higher the AUC, the better the performance of the model in distinguishing between two classes. Compared to accuracy, the AUC is a better performance metric (Huang and Ling 2005).

Furthermore, we repeated with selection of links from the training sets based on the top 15%, 20%, 30%, 40%, to 100% of the significantly different functional connectivities, to investigate how selecting more functional connectivities from the training set might lead to better prediction when the SVM was applied to the links in the test set.

### Replication investigation with Human Connectome Project data

To investigate whether the findings were also present in a group with a much younger age, resting state fMRI data at 3T from 1000 individuals (532 females) in the Human Connectome Project (Smith et al. 2013) were analysed, with the general methods described previously (Feng et al. 2020; Ma et al. 2022). The mean age was 28.7 years (sd 3.7). A *t*-test was performed to test whether there were differences in the functional connectivity matrices for the 360 cortical areas in the HCP-MMP atlas (Glasser et al. 2016a; Huang et al. 2022), using the same covariates as previously (Feng et al. 2020) namely age, Townsend deprivation index (which includes information for example about the loss of parents), frequency of drinking alcohol, smoking status, educational qualifications, and head motion (mean Framewise Displacement).

## Results

**Fig. 1:**
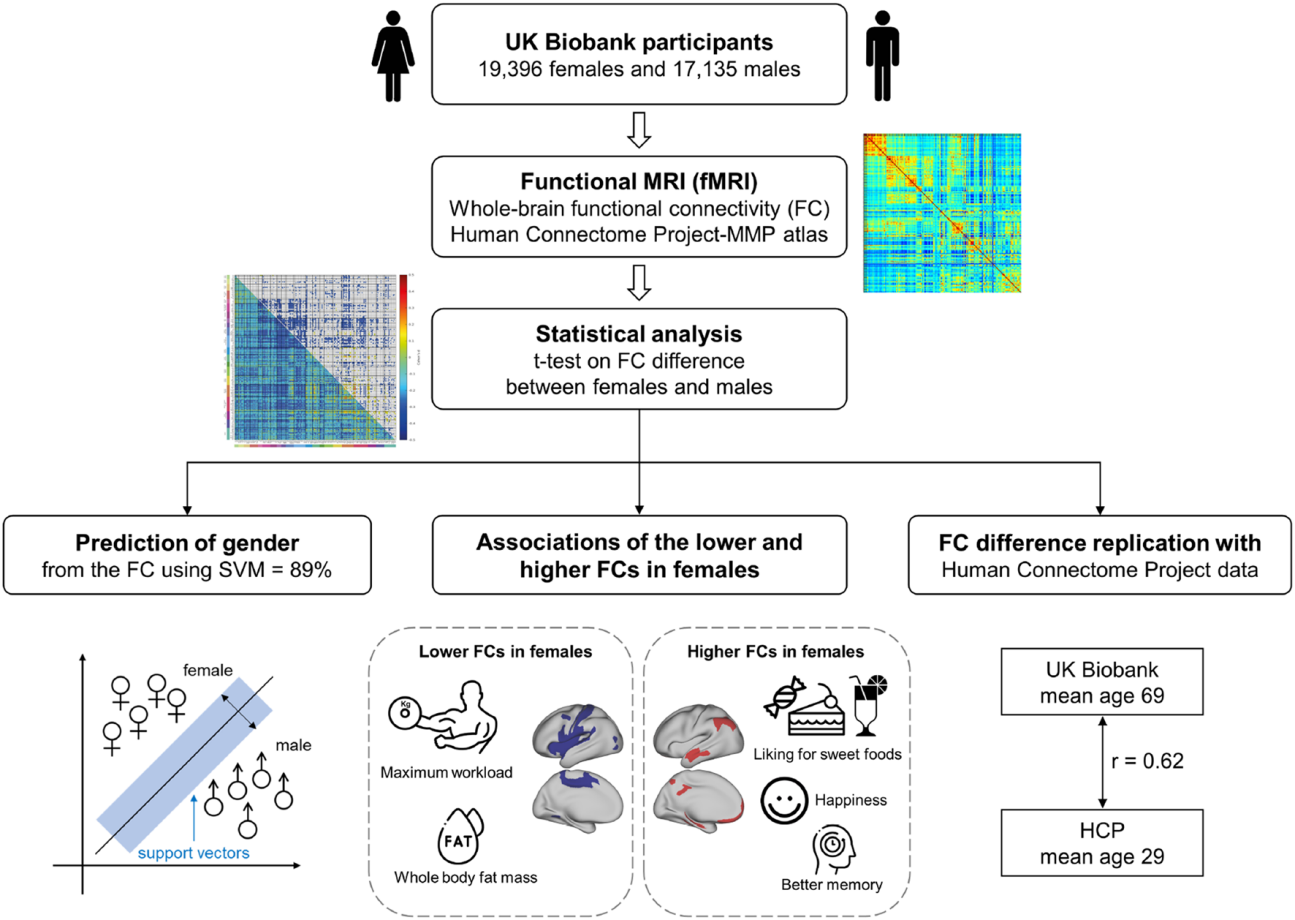
Workflow and summary of findings. Functional connectivity was calculated, and differences for females minus males were calculated with Bonferroni correction for multiple comparisons. Association analyses with measures in the UK Biobank showed that many of the lower functional connectivities in females were for somatosensory/motor cortical regions, and were correlated with lower maximal workload and higher body fat mass. The higher functional connectivities in females were correlated with higher liking for sweet food, happiness, and better memory scores

The workflow, and an overview of some of the findings, are shown in Fig. 1.

### Functional Connectivity differences between females and males

**Fig. 2:**
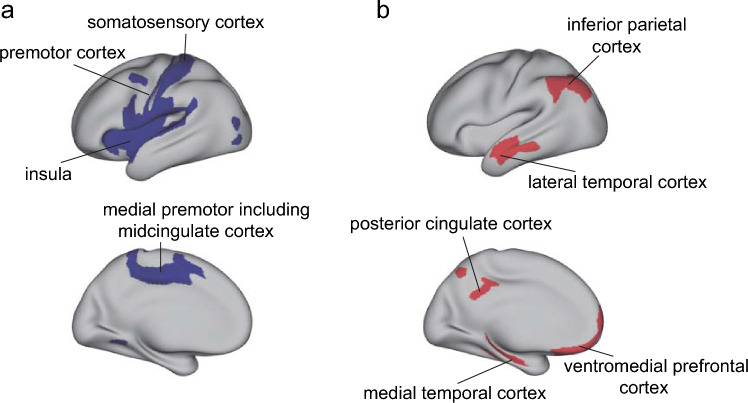
The main cortical regions with lower (**a**) or higher (**b**) functional connectivity in females (*n* = 19,396) compared to males (*n* = 17,135). The regions in the HCP-MMP atlas shown in color are from the results shown in Fig. 3, and were selected by showing cortical regions for which the number of significant links was in the top 25%

**Fig. 3:**
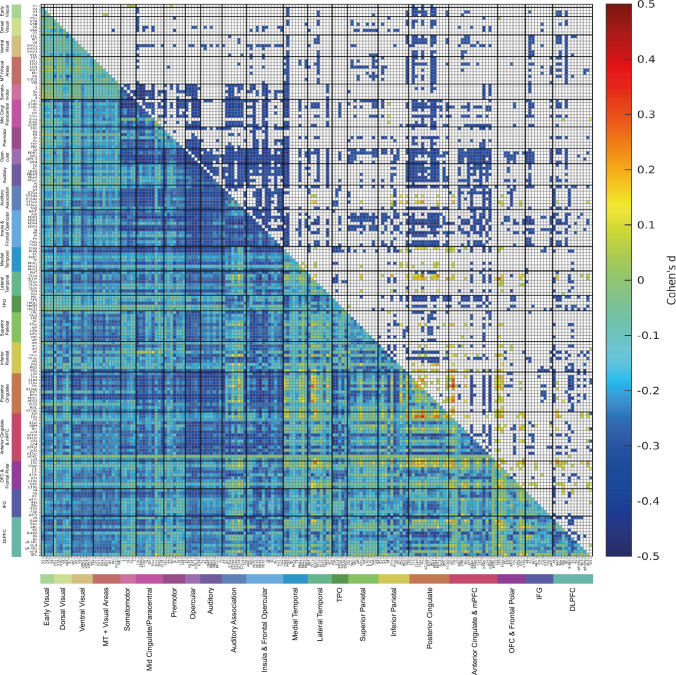
The lower left triangle shows the matrix of functional connectivity differences for females—males with the Cohen’s *d* values showing the effect size of the differences. The matrix is for the functional connectivities in the left hemisphere, as listed in Table S1, with V1, V2, V3 … at the top of the y axis and the left of the x axis. The upper triangle matrix shows the Cohen’s *d* values of the top 20% significant negative links and all significant positive links after Bonferroni correction (corrected *p* < 3.1e^−6^). These results were from 19,396 females and 17,135 males. The negative values shown in the upper right triangle had *d* <  − 0.29, and all the values shown in the matrix were in the range from − 0.5 to 0.5. The standard covariates regressed out in this analysis were Age, BMI, education qualifications, smoking status, drinker status, Townsend deprivation index, head motion, and imaging site information. The cortical regions in the HCP-MMP atlas are illustrated in Figs. S1 and S2, and their names and divisions are shown in Table S1. The cortical divisions are separated by thick lines, and labelled at the bottom of the figure

To find how the functional connectivity of female and male brains is different, two-sample *t*-tests were performed to compare each functional connectivity link in the Human Connectome Project extended Multimodal Parcellation atlas (Glasser et al. 2016a; Huang et al. 2022) functional connectivity matrices for females vs males, using Bonferroni correction for multiple comparisons, with many covariates regressed out as described in the Methods. The functional connectivity differences between females (*n* = 19,396) and males (*n* = 17,135) are summarized in Fig. 2, and are shown in terms of effect size measured by Cohen’s d in detail as functional connectivity matrices in Fig. 3 for the 180 cortical regions in the left hemisphere, and in Fig. S3 for the right hemisphere. Figure 2a shows that the cortical regions with many lower functional connectivities in females compared to males included many somatosensory and premotor cortical regions. Figure 2b shows that the cortical regions with higher functional connectivities in females compared to males included regions in the ventromedial prefrontal cortex, lateral temporal cortex, posterior cingulate cortex and medial temporal cortex, and the inferior parietal cortex.

Figure 3 shows that after Bonferroni correction for multiple comparisons given that the $\frac{180\times (180-1)}{2}$ links in the HCPex atlas are being compared, most links were significantly different (corrected *p* < 3.1 × 10^–6^, even with the regression out of the effects of covariates of no interest listed in the Methods). Figure 3 therefore shows only the top 20% of significantly different functional connectivities in the top right triangle, to enable some of the larger differences to be assessed. The effect sizes measured by Cohen’s *d* were reasonably moderate in size, typically greater than $\pm 0.3$, as shown in Fig. 3. The mean functional connectivity difference across all 180 cortical regions in the left hemisphere was lower in females than males, with Cohen’s *d* = −0.18. The lower functional connectivities in females shown in Fig. 3 involved especially divisions in the HCP-MMP atlas involved in somatomotor function (see Rolls et al. (2023d)), including the Somatomotor, Midcingulate and Paracentral, Premotor, Opercular, and Insular and Frontal Opercular divisions. As shown in Fig. 3, this set of cortical regions had in females lower connectivity with each other, but also with inferior parietal division regions involved in somatosensory representations (Rolls et al. 2023d, e), cortical regions in the posterior cingulate division involved in episodic memory which has spatial components (Rolls et al. 2023b), and with regions in the Anterior Cingulate and Medial Prefrontal Division including the supracallosal parts of the anterior cingulate cortex with somatomotor connectivity implicated in action–outcome learning (Rolls et al. 2023c). Another set of regions with lower functional connectivity in females involved regions in the Auditory Association Division, especially those involving the cortex in the superior temporal sulcus (STS), which are involved in semantic including auditory but also visual representations (Rolls et al. 2022a, 2023a; Rolls et al. 2023a). The lower connectivities of these STS cortical regions in females were especially with the somatosensory regions just described (Fig. 3). To provide more evidence about these differences, the top 50 functional connectivity links that were more negative in females are shown in Table S2. The connectivity differences between females and males were similar for the right hemisphere, as shown in Fig. S3. Moreover, as shown in Fig. S7, very interestingly, the functional connectivity differences between females and males within each hemisphere were also in general found also for the connectivities between the two hemispheres.

Though most of the functional connectivities were lower in females, the differences of functional connectivity between females and males were not uniform across all cortical regions, and indeed as shown in Figs. 2b and 3 links involving the episodic memory related posterior cingulate cortex (Rolls et al. 2023b) (especially with inferior temporal cortex TE1a and TE1m and cortex in the superior temporal sulcus implicated in visual and semantic processing (Rolls et al. 2022a, 2023a)), and the ventromedial prefrontal cortex including 10r, 10v 10d and also 10 pp (Rolls 2022; Rolls et al. 2023c) (especially with the posterior cingulate cortex) were higher in females. To provide more evidence about these differences, the top 50 functional connectivity links that were more positive in females are shown in Table S3. The top 50 links that had higher functional connectivities in females included 30 links involving the posterior cingulate cortex, 21 links involving the anterior cingulate and ventromedial prefrontal cortex, 18 links involving the orbitofrontal and frontal pole cortex, 11 links involving the lateral temporal cortex, and 10 links involving the inferior parietal cortex (see also Fig. 2).

To ensure that these results were not due to differences in total intracranial volume between females and males, we repeated the analysis shown in Fig. 3 with total intracranial volume regressed out, and found that this made essentially no difference, with the correlation for the Cohen’s d matrix between the two analyses 0.95.

It is noted that many of the functional connectivities were significantly different between females and males in this large scale study, which has considerable sensitivity because of the large numbers of participants, and it is to facilitate interpretation that only the top 20% of the significantly different links that were lower in females are shown in the top right triangle of Fig. 3. Also to facilitate interpretation of the effects, the effect size measured by Cohen’s d is what is provided in Fig. 3.

### Prediction of sex from the functional connectivity using a Support Vector Machine

**Fig. 4:**
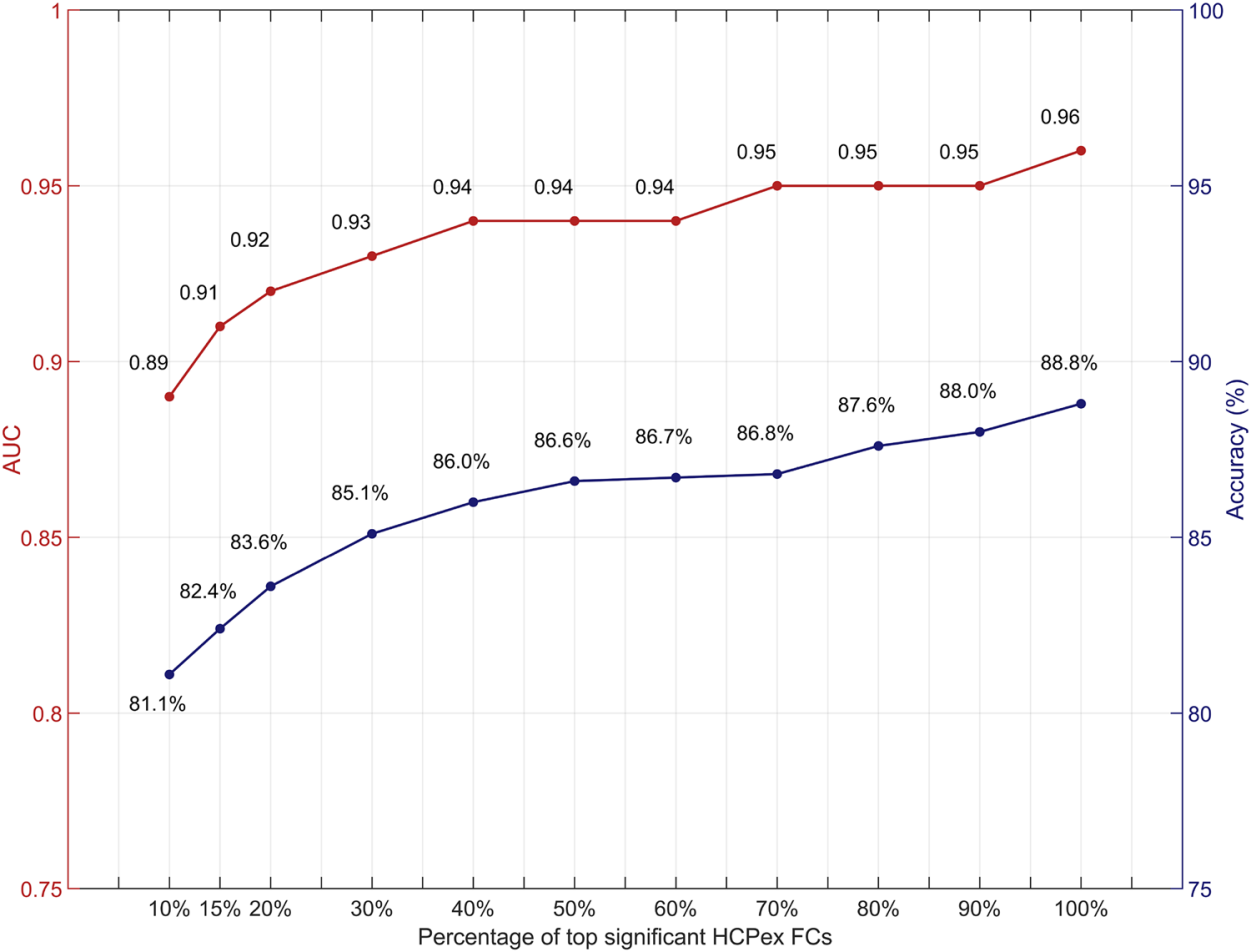
Prediction of sex from the functional connectivity using SVM. AUCs and the accuracy of the sex classification based on the functional connectivities in the HCPex atlas which were used as the features to train the model. The functional connectivity links for this test were selected from the top percentage of significant links after Bonferroni correction shown on the x axis in the training set. The results are shown for prediction in the group not used for training in the split data design. The accuracy and AUC increased as more of the significantly different links between females and males were used

The support vector machine analysis showed that sex could be predicted from the functional connectivity with the best performance at 88.8% correct and an AUC of 0.96 (Fig. 4). The best performance was when 100% of the significantly different links between females and males in the training (discovery) set were used to identify the links to be used for the SVM analysis in the test set (Fig. 3). (The AUC measure lies between 0 and 1, with 0.5 representing chance performance.)

### Effect of covariates on the differences of functional connectivity difference between females and males

The standard covariates regressed out in this analysis shown in Fig. 3 were age, BMI, education qualifications, smoking status, drinker status, Townsend deprivation index, head motion, and imaging site information. To investigate what possible factors might be related to the differences of functional connectivity in females—males, we investigated the hypotheses specified in the Introduction. The *t*-test results showed that the Maximum workload during the fitness test was lower in females than males (65.4 vs 97.6 watts, *t* = –43.3, *p* < 1.0 × 10^–15^, *n* = 2147 females, 1940 males). We also found that the whole body fat mass was greater in females than males (25.6 vs 21.5 kg, *t* = 59.7, *p* < 1.0 × 10^–15^, *n* = 2147 females, 1940 males. In the analyses, we controlled covariates including age, BMI, education qualifications, smoking status, drinker status, and Townsend deprivation index.

**Fig. 5:**
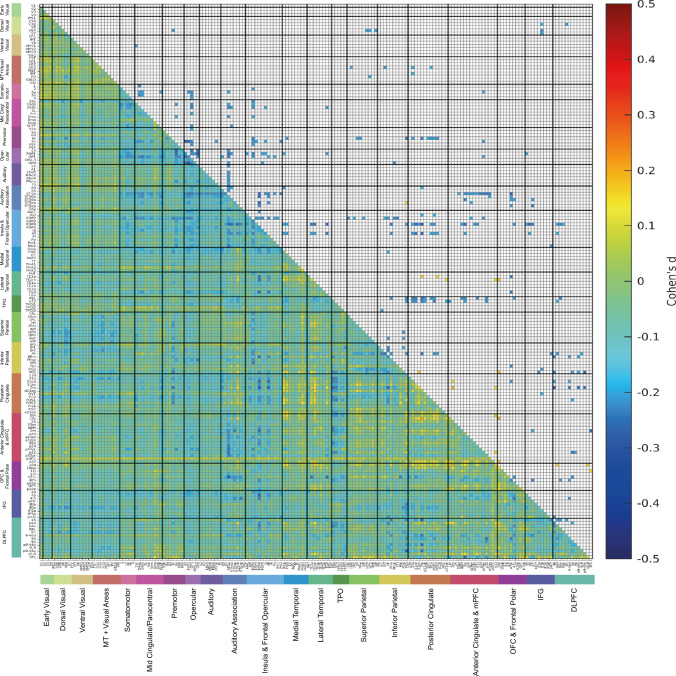
The lower left triangle shows the matrix of functional connectivity differences for females—males with the Cohen’s *d* values showing the effect size of the differences. The standard covariates were regressed out, but also so too were Field 6032 Maximum workload during fitness test and Field 23,100 Whole body fat mass as covariates in the two-sample *t*-tests. The matrix is for the functional connectivities in the left hemisphere, as listed in Table S1, with V1, V2, V3 … at the top of the y axis and the left of the x axis. The upper triangle matrix shows the Cohen’s d values of top 20% significant negative links and all significant positive links after Bonferroni correction (corrected *p* < 3.1e^−6^). These results were from 2099 females and 1889 males. All the values shown in the matrix were in the range from − 0.5 to 0.5

Given that many of the lower functional connectivities in females were for somatosensory / premotor regions (Figs. 2 and 3), it was hypothesized that these two variables might relate to the functional connectivity differences across the whole cortex in females vs males. To test this hypothesis, we added these two variables as additional covariates in the analysis of sex differences in functional connectivity, and found the results shown in Fig. 5 and Table 2. Figure 5 makes it clear that with both additional covariates used, many fewer links had large differences in functional connectivity. (A comparison for exactly the same participants without these two additional covariates regressed out is shown in Fig. S4, which is very similar to what is shown in Fig. 3.) The results shown in Table 2 make it clear that the mean Cohen’s *d* across all FC links for the difference of functional connectivity for females—males reduced from –0.18 to –0.06 when these two covariates are added, with total body fat mass being more involved than the workload measure. A paired *t*-test showed that the mean value across participants for the Cohen’s d matrix in Fig. 5 was significantly greater than that of Fig. S4 (*t* = 254.0, *p* < 1.0 × 10^–15^). This indicated that the difference of FC for females—males in the functional connectivity across the whole cortex was greatly reduced by regressing out these two covariates, maximum workload and whole body fat mass.

**Table 2:** Effect of regressing out additional variables on the effect size for the mean difference of FC for females—males

Covariates	Mean of the *d* values of whole-brain links	Mean of the d values of links related to somatosensory/premotor	Mean of the d values of the significantly positive links in Fig. S4 which includes only the standard covariates
Standard covariates only	− 0.18	− 0.20	0.20
Standard covariates + Maximum workload during fitness test	− 0.12	− 0.13	0.16
Standard covariates + Whole body fat mass	− 0.10	− 0.12	0.14
Standard covariates + Maximum workload during fitness test AND Whole body fat mass	− 0.06	− 0.07	0.12

### Different functional connectivities between females and males relate to body and behavioral characteristics

Evidence that the higher functional connectivities of the somatosensory regions in males are related to the behavioral measures such as the higher strength of males and their lower whole body fat mass was sought by measuring the partial correlations between these functional connectivities and the previously hypothesized behavioral measures, with regression out of the same covariates of no interest described above. After Bonferroni correction (0.05/5), a highly significant correlation was found across the whole population between the mean of the functional connectivities in somatosensory cortex regions 1-OP4 in the HCP-MMP atlas and the Maximum workload during fitness test (Field 6032): *r* = 0.17, *p* = 1.59 × 10^–28^ (*n* = 3988). Moreover, a highly significant correlation was also found across the whole population between the mean of the functional connectivities in somatosensory cortex regions 1-OP4 and whole body fat mass (Field23100): *r* = –0.17, *p* = 1.10 × 10^–28^ (*n* = 3988). This provides an indication that the lower functional connectivities of the somatosensory regions in females are related to their lower physical strength and higher body fat mass across the population.

In addition, factors correlated with the significantly higher functional connectivities in females were analysed. It was found that after Bonferroni correction (0.05/5), the mean functional connectivity of links significantly higher in females in Fig. 3 was positively correlated with the liking for sweet foods (the mean from category 1039 of apple juice, biscuits, cake, icing, honey, chocolate, orange juice and liking for sweet foods) (*r* = 0.02, *p* = 0.006); with category happiness / well-being (*r* = 0.02, *p* = 0.01); and with the cognitive task Tower rearranging (which probably has a memory requirement) (*r* = 0.03, *p* = 1.2 × 10^–4^). These results are for all links that were significantly higher in females. Details of how these behavioral measures had higher correlations with some of the links that were higher in females are provided in Figs. S5-S6.

Consistent with these associations, it was found in this dataset that the liking for sweet foods was higher in females (6.2 vs 5.9, *t* = 11.2, *p* = 3.4 × 10^–29^, *n* = 17,088 females, 14,465 males); and prospective memory was higher in females (1.11 vs 1.08, *t* = 4.0, *p* = 6.6 × 10^–5^, *n* = 6664 females, 6048 males).

## Replication investigation with Human Connectome Project data

**Fig. 6:**
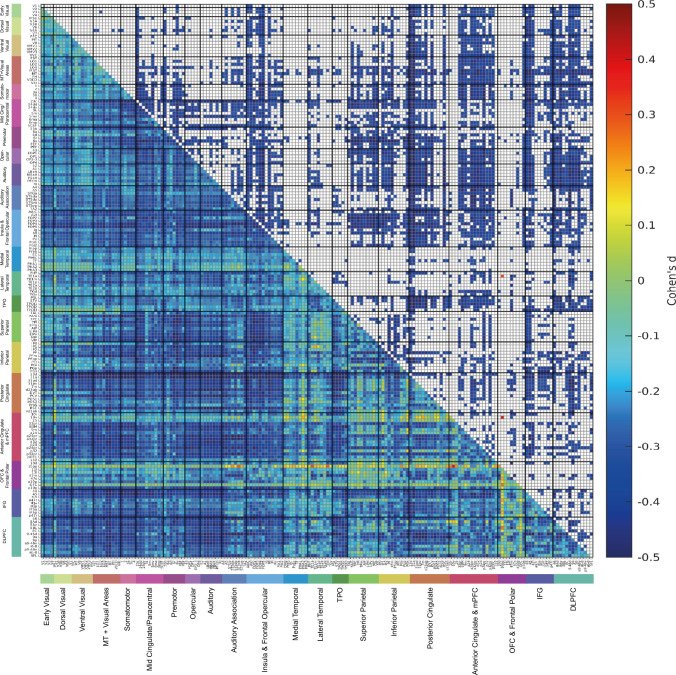
Differences in resting state functional connectivity in females—males with data from 1000 individuals (532 females) in the Human Connectome Project. The lower left triangle shows the Cohen’s d values for the differences, and the upper right triangle shows the differences that were significant after Bonferroni correction for the number of comparisons made in the $180\times 180$ functional connectivity matrices for the left hemisphere. Negative values in this matrix indicate lower functional connectivities in females. All the values shown in the matrix were in the range from − 0.5 to 0.5. The conventions are as in Fig. 3

To investigate whether the findings were also present in a group with a much younger age, resting state fMRI data at 3T from 1000 individuals (532 females) in the Human Connectome Project (Smith et al. 2013) were analysed, with the general methods described previously (Feng et al. 2020; Ma et al. 2022). The mean age was 28.7 years (sd 3.7). Figure 6 shows the results for a t-test for whether there were differences in the functional connectivity matrices for the 180 cortical areas in the left hemisphere in the HCP-MMP atlas (Glasser et al. 2016a; Huang et al. 2022), using the same covariates as previously (Feng et al. 2020). Similar results were obtained for the right hemisphere. The results were similar to those in the UK Biobank, in that the two *t*-differences matrices (from the UK Biobank and the HCP) were correlated 0.62.

As shown in Fig. 6, the effect sizes were considerable, with the Cohen’s d for many of the significantly different links in the range –0.3 to –0.5.

The main features of the differences in functional connectivity were as follows for the HCP dataset. First, the mean functional connectivity across all cortical regions in the left hemisphere was lower in females than males (*t* = –6.80, *p* = 1.85 × 10^–11^, Cohen’s *d* = –0.43). Second the lower functional connectivities in females were especially for cortical regions that included the somatosensory / premotor cortical regions including the frontal opercular (FOP) and insular regions (Rolls et al. 2023d); for the posterior cingulate division in the HCP-MMP atlas (see Table S1) (Glasser et al. 2016a; Huang et al. 2022; Rolls et al. 2023e); for much of the anterior cingulate cortex (Rolls et al. 2023c); for Broca’s area (44, 45 and 47l) (Rolls et al. 2022a); for dorsolateral prefrontal cortex and dorsal prefrontal cortex regions implicated in short-term memory and attention (Rolls et al. 2023d); and for some intraparietal and parietal area 7 regions (Rolls et al. 2023e). There were few significant differences in the functional connectivities between females and males for visual cortical regions (Fig. 6). Consistent with what was found in the UK Biobank, for the HCP some functional connectivities were higher than the average in females, including for frontal pole 10pp, orbitofrontal and vmPFC (OFC, 10v), and parahippocampal PHA2 regions (Fig. 6).

## Discussion

First, we found that functional connectivities were overall lower in the brains of females than males, but in a much larger sample than was used previously, and with a much more detailed cortical atlas (Zhang et al. 2016).

Second, use of the HCP-MMP atlas (Glasser et al. 2016a; Huang et al. 2022) enabled us to show that many of the lower functional connectivities in females were related to somatosensory / motor cortical areas, greatly extending previous research (Ritchie et al. 2018) by identifying the cortical regions involved much more precisely using the HCP-MMP atlas. These regions included many in the Somatomotor, Midcingulate and Paracentral, Premotor, Opercular, and Insular and Frontal Opercular divisions of the HCP-MMP atlas (Huang et al. 2022). This set of cortical regions had in females lower connectivity primarily with each other, but also with inferior parietal division regions involved in somatosensory representations (Rolls et al. 2023d, e), cortical regions in the posterior cingulate division involved in episodic memory which has spatial components (Rolls et al. 2023b), and with regions in the Anterior Cingulate and Medial Prefrontal Division including the supracallosal parts of the anterior cingulate cortex with somatomotor connectivity implicated in action-reward outcome learning (Rolls 2023b; Rolls et al. 2023c). Another set of regions with lower functional connectivity in females involved regions in the Auditory Association Division, especially those involving the cortex in the superior temporal sulcus (STS), which are involved in semantic including auditory but also visual representations (Rolls et al. 2022a, 2023a; Rolls et al. 2023a). The lower connectivities of these STS cortical regions were especially with the somatosensory regions just described (Fig. 3). An implication is that the connectivities of somatomotor regions that are lower in females include connectivities with semantic cortical regions involved in language (Rolls et al. 2022a), and that may have some implications for cognitive function.

Third, we were able to follow up the implications of the somatomotor differences in functional connectivity between females and males, and were able to show not only that many of these lower functional connectivities in females were correlated with physical differences between females and males such as maximum physical workload (lower in females) and body fat mass (higher in females), but also that regressing out the effects of these two physical variables greatly reduced the mean difference across all functional connectivities in females (from *d* =  − 0.18 to − 0.06). Moreover, a highly significant correlation was found between the mean of the functional connectivities in somatosensory cortex regions 1-OP4 in the HCP-MMP atlas and the Maximum workload during fitness test (*r* = 0.17, *p* = 1.59 × 10^–28^); and whole body fat mass (*r* =  − 0.17, *p* = 1.10 × 10^–28^). We provide in this way for the first time clear evidence that the lower functional connectivities of the somatosensory regions in females are related to their lower physical strength and higher body fat mass across the population. The direction of the causality here is unclear, but one possibility is that the different motor and sensory functioning on average in males due to lifestyle leads to increased functional connectivities in some somatosensory/motor cortical regions. It is well established that different sensory-motor engagement can lead to altered functioning of cortical regions (Kolb and Gibb 2014). Alternatively, it could be that genetic specification of differences between females and males includes a neuromotor system in males that is adapted for motor function including strength. We note that possible confounders such as BMI did not contribute to this sex difference, as this (and many other measures) were regressed out, as described in the Methods.

Fourth, the differences in functional connectivity between females and males were not uniform across all cortical regions, and functional connectivities involving the ventromedial prefrontal cortex including 10r, 10v 10d and also 10 pp, cortical regions involved in reward and emotion (Rolls 2022; Rolls et al. 2023c) were higher in females, especially for links with the posterior cingulate cortex. In addition, functional connectivities of the posterior cingulate cortex, which is implicated in episodic memory (Rolls et al. 2023b) (especially with the medial temporal lobe regions implicated in episodic memory Rolls et al. 2022b; Rolls 2023a, c), anterior inferior temporal cortex TE1a and TE1m and inferior parietal PGs and PGi implicated in semantic functions (Rolls et al. 2022a, 2023e)), were higher in females. These higher functional connectivities in females were positively correlated with the liking for sweet foods (e.g., apple juice, biscuits, cake, icing, honey, chocolate and orange juice). The higher connectivities of these cortical regions associated with some types of food reward clearly may relate to food preferences in females, and in this population, the liking for sweet foods was indeed higher in the females (see Results). The differences identified here in the brains of females may extend to other types of reward behaviors in females that are likely to be implemented by these brain regions (Rolls 2018, 2019; Rolls et al. 2020; Rolls 2023b; Rolls et al. 2023c) than are measured in the UK Biobank. Indeed, it was found in the population analysed here that ‘happiness and subjective well-being’ were higher in the females.

The higher functional connectivity in females of the posterior cingulate cortex identified here is also of interest, for this region is implicated in episodic memory (Rolls et al. 2023b), and so is the ventromedial prefrontal cortex / anterior cingulate cortex (Bonnici and Maguire 2018; McCormick et al. 2018; Ciaramelli et al. 2019). Indeed, in this large sample, prospective memory was higher in females than males. The ventromedial prefrontal and anterior cingulate cortex regions may relate to episodic memory because of the connectivity of these brain regions to the hippocampus (Rolls 2022; Rolls et al. 2023c), and to the septum which contains cholinergic neurons that innervate the hippocampus (Rolls 2022; Rolls et al. 2023c) and are involved in memory consolidation (Hasselmo and Sarter 2011; Newman et al. 2012; Rolls 2022). An implication is that some types or aspects of episodic memory, perhaps including autobiographical memory, may be better in females. Consistent with this, sex differences in memory have been reported in a number of studies (Asperholm et al. 2019). In general, females have an advantage in verbal memory (Voyer et al. 2007; Stoet and Geary 2018; Voyer et al. 2021). Another meta-analysis based on 355,173 participants provided evidence that females outperformed males in verbal episodic memory (Hirnstein et al. 2022). An fMRI study found that during an autobiographical memory test, females showed higher BOLD activity in the right dorsolateral prefrontal cortex, left dorsal anterior insula and right precuneus (Young et al. 2013). In addition, greater female superiority in object location memory tasks has also been described (Voyer et al. 2017). The vmPFC and connected regions may be involved in memory because these regions introduce reward information into the hippocampal memory system, and because these vmPFC regions have connectivity to the cholinergic systems that are involved in memory consolidation (Rolls 2022). The research described here indicates that the difference in episodic and autobiographical memory in females may be related to the higher functional connectivity of the posterior cingulate cortex with cortical regions such as the vmPFC, medial temporal cortical regions, anterior temporal regions, and inferior parietal regions in females (Fig. 3), as shown here.

In conclusion, this investigation shows for the first time with the HCP-MMP atlas which cortical systems have different functional connectivity between females and males. Moreover, for the first time we related these differences of functional connectivity to particular physical and behavioral measures, showing that the lower functional connectivity of somatomotor cortical systems in females relates across the population to lower physical strength and higher body fat mass; and that the higher functional connectivities in females of vmPFC and posterior cingulate cortex regions relate to greater liking in females of sweet foods, to greater feelings of well-being in females, and to better performance on a memory-intensive cognitive task.

### Supplementary Information

Below is the link to the electronic supplementary material.

Supplementary file1 (DOCX 8196 KB)

## Data Availability

The data analyzed are available from the UK Biobank (https://biobank.ctsu.ox.ac.uk), and from the HCP website http://www.humanconnectome.org/. Standard Matlab functions were used to calculate the functional connectivity, to perform the Bonferroni corrections for multiple comparisons, and to perform the prediction analyses using a Support Vector Machine (Statistics and Machine Learning Toolbox Release 2021b, The MathWorks, Inc., Natick, Massachusetts, United States). The HCPex atlas (Huang et al. 2022) is available at https://www.oxcns.org.
