# Supplementary material for: Different cortical connectivities in human females and males relate to differences in strength and body composition, reward and emotional systems, and memory
Source: Brain Struct Funct. 2023 Oct 20;229(1):47–61. doi: 10.1007/s00429-023-02720-0 (PMC10827883; doi:10.1007/s00429-023-02720-0)
Supplement: Supplementary file 1 — Supplementary file1 (DOCX 8196 KB) [file 429_2023_2720_MOESM1_ESM.docx]

**Different cortical connectivities in human females and males relate to differences in strength and body composition, reward and emotional systems, and memory**

**Supplementary Material**

**Brain Structure and Function (2023)** doi: 10.1007/s00429-023-02720-0

Ruohan Zhang^1^, Edmund T Rolls^1,2,3^, Wei Cheng^2^ and Jianfeng Feng^1,2^

1. Department of Computer Science, University of Warwick, Coventry, CV4 7AL, UK
2. Institute of Science and Technology for Brain Inspired Intelligence, Fudan University, Shanghai 200403, China
3. Oxford Centre for Computational Neuroscience, Oxford, UK

*Corresponding author information:

Professor Edmund T. Rolls,

Department of Computer Science, University of Warwick, Coventry CV4 7AL, UK. Email: [Edmund.Rolls@oxcns.org](mailto:Edmund.Rolls@oxcns.org)

URL: [https://www.oxcns.org](https://www.oxcns.org/)

<https://orcid.org/0000-0003-3025-1292>

**Modified ordering of the HCP-MMP atlas**

The atlas used to define brain regions was the HCP-MMP surface-based atlas (Glasser, et al., 2016), illustrated in Figs. S1 and S2. In the HCP-MMP atlas, each region has its RegionID, which we show in Table S1. Detailed information about the regions is available in the Supplementary Material File NIHMS68870-supplement-Neuroanatomical_Supplementary_Results.pdf provided by Glasser et al (2016). In that Supplementary Material file, a grouping of the regions is suggested based on geographic proximity and functional similarities, and this grouping is shown in the column labelled CortexID in Table S1. That has led to a different ordering of the regions, which we show in Table S1, with the original regionIDs from the HCP atlas shown in the column headed ‘regionID’. This reordered version of the HCP-MMP atlas is described by Dr Dianne Patterson of the University of Arizona at <https://neuroimaging-core-docs.readthedocs.io/en/latest/pages/atlases.html>, where the following supporting files used to help generate Table S1 are available: HCP-MMP_UniqueRegionList.csv and Glasser_2016_Table.xlsx. We made file HCPMMP_CortexID_Ordering.xlsx from this, and this is available from the present authors. The connectivity matrices shown in the present paper used the ordering shown in Table S1, which is also used in the volumetric and extended form of this atlas (Huang, et al., 2022).

**Table S1.** Regions defined in the modified Human Connectome Project atlas (Glasser, et al., 2016). L=left hemisphere, R=right. The column ‘Reordered region ID’ is that used in Figs.3, 5, 6, and is a reordering of that based on suggestions in the Supplementary Information of Glasser et al (2016). In that Supplementary Information of that paper, the 360 regions are grouped based on geographic proximity and functional similarities, which was reorganized and provided by Dr Dianne Patterson of the University of Arizona at [https://neuroimaging-core-docs.readthedocs.io/en/latest/pages/atlases.html](https://neuroimaging-core-docs.readthedocs.io/en/latest/pages/atlases.html%20) with the HCP-MMP_UniqueRegionList.csv and is shown in the column labelled CortexID in Table S1. The volumes are in mm^3^. This modified atlas with the reordering is described elsewhere (Huang, et al., 2022).

| **Reordered**  **ID (L, R)** | **Region** | **RegionLongName** | **Cortical Division** | **Cortex**  **ID** | **Original**  **ID** | **Voxel numbers (1mm^3^) (L,R)** |
| --- | --- | --- | --- | --- | --- | --- |
| 1, 181 | V1 | Primary_Visual_Cortex | Primary_Visual | 1 | 1 | 13812, 13406 |
| 2, 182 | V2 | Second_Visual_Area | Early_Visual | 2 | 4 | 9515, 9420 |
| 3, 183 | V3 | Third_Visual_Area | Early_Visual | 2 | 5 | 7106, 7481 |
| 4, 184 | V4 | Fourth_Visual_Area | Early_Visual | 2 | 6 | 4782, 4537 |
| 5, 185 | IPS1 | IntraParietal_Sulcus_Area_1 | Dorsal_Stream_Visual | 3 | 17 | 1751, 1750 |
| 6, 186 | V3A | Area_V3A | Dorsal_Stream_Visual | 3 | 13 | 2191, 2212 |
| 7, 187 | V3B | Area_V3B | Dorsal_Stream_Visual | 3 | 19 | 639, 731 |
| 8, 188 | V6 | Sixth_Visual_Area | Dorsal_Stream_Visual | 3 | 3 | 1402, 1559 |
| 9, 189 | V6A | Area_V6A | Dorsal_Stream_Visual | 3 | 152 | 904, 734 |
| 10, 190 | V7 | Seventh_Visual_Area | Dorsal_Stream_Visual | 3 | 16 | 1005, 1041 |
| 11, 191 | FFC | Fusiform_Face_Complex | Ventral_Stream_Visual | 4 | 18 | 3848, 4402 |
| 12, 192 | PIT | Posterior_InferoTemporal_complex | Ventral_Stream_Visual | 4 | 22 | 1392, 1386 |
| 13, 193 | V8 | Eighth_Visual_Area | Ventral_Stream_Visual | 4 | 7 | 1361, 1175 |
| 14, 194 | VMV1 | VentroMedial_Visual_Area_1 | Ventral_Stream_Visual | 4 | 153 | 939, 1219 |
| 15, 195 | VMV2 | VentroMedial_Visual_Area_2 | Ventral_Stream_Visual | 4 | 160 | 639, 923 |
| 16, 196 | VMV3 | VentroMedial_Visual_Area_3 | Ventral_Stream_Visual | 4 | 154 | 941, 1242 |
| 17, 197 | VVC | Ventral_Visual_Complex | Ventral_Stream_Visual | 4 | 163 | 2487, 2753 |
| 18, 198 | FST | Area_FST | MT+_Complex | 5 | 157 | 1324, 1683 |
| 19, 199 | LO1 | Area_Lateral_Occipital_1 | MT+_Complex | 5 | 20 | 619, 909 |
| 20, 200 | LO2 | Area_Lateral_Occipital_2 | MT+_Complex | 5 | 21 | 1179, 1062 |
| 21, 201 | LO3 | Area_Lateral_Occipital_3 | MT+_Complex | 5 | 159 | 438, 915 |
| 22, 202 | MST | Medial_Superior_Temporal_Area | MT+_Complex | 5 | 2 | 794, 1036 |
| 23, 203 | MT | Middle_Temporal_Area | MT+_Complex | 5 | 23 | 620, 1005 |
| 24, 204 | PH | Area_PH | MT+_Complex | 5 | 138 | 3453, 3205 |
| 25, 205 | V3CD | Area_V3CD | MT+_Complex | 5 | 158 | 876, 1222 |
| 26, 206 | V4t | Area_V4t | MT+_Complex | 5 | 156 | 1037, 1249 |
| 27, 207 | 1 | Area_1 | SomaSens_Motor | 6 | 51 | 6590, 5925 |
| 28, 208 | 2 | Area_2 | SomaSens_Motor | 6 | 52 | 4278, 4727 |
| 29, 209 | 3a | Area_3a | SomaSens_Motor | 6 | 53 | 2247, 2286 |
| 30, 210 | 3b | Primary_Sensory_Cortex | SomaSens_Motor | 6 | 9 | 5451, 4350 |
| 31, 211 | 4 | Primary_Motor_Cortex | SomaSens_Motor | 6 | 8 | 10776, 10254 |
| 32, 212 | 23c | Area_23c | ParaCentral_MidCing | 7 | 38 | 2259, 2498 |
| 33, 213 | 24dd | Dorsal_Area_24d | ParaCentral_MidCing | 7 | 40 | 2665, 2820 |
| 34, 214 | 24dv | Ventral_Area_24d | ParaCentral_MidCing | 7 | 41 | 1076, 1349 |
| 35, 215 | 5L | Area_5L | ParaCentral_MidCing | 7 | 39 | 2249, 2327 |
| 36, 216 | 5m | Area_5m | ParaCentral_MidCing | 7 | 36 | 1483, 2079 |
| 37, 217 | 5mv | Area_5m_ventral | ParaCentral_MidCing | 7 | 37 | 1651, 1996 |
| 38, 218 | 6ma | Area_6m_anterior | ParaCentral_MidCing | 7 | 44 | 3941, 4251 |
| 39, 219 | 6mp | Area_6mp | ParaCentral_MidCing | 7 | 55 | 3701, 3105 |
| 40, 220 | SCEF | Supplementary_and_Cingulate_Eye_Field | ParaCentral_MidCing | 7 | 43 | 3500, 3371 |
| 41, 221 | 55b | Area_55b | Premotor | 8 | 12 | 2422, 1537 |
| 42, 222 | 6a | Area_6_anterior | Premotor | 8 | 96 | 4233, 3752 |
| 43, 223 | 6d | Dorsal_area_6 | Premotor | 8 | 54 | 2916, 2909 |
| 44, 224 | 6r | Rostral_Area_6 | Premotor | 8 | 78 | 3029, 3981 |
| 45, 225 | 6v | Ventral_Area_6 | Premotor | 8 | 56 | 2075, 2516 |
| 46, 226 | FEF | Frontal_Eye_Fields | Premotor | 8 | 10 | 1787, 1889 |
| 47, 227 | PEF | Premotor_Eye_Field | Premotor | 8 | 11 | 1006, 1258 |
| 48, 228 | 43 | Area_43 | Posterior_Opercular | 9 | 99 | 1889, 1678 |
| 49, 229 | FOP1 | Frontal_Opercular_Area_1 | Posterior_Opercular | 9 | 113 | 879, 932 |
| 50, 230 | OP1 | Area_OP1-SII | Posterior_Opercular | 9 | 101 | 1275, 1072 |
| 51, 231 | OP2-3 | Area_OP2-3-VS | Posterior_Opercular | 9 | 102 | 943, 792 |
| 52, 232 | OP4 | Area_OP4-PV | Posterior_Opercular | 9 | 100 | 2332, 2409 |
| 53, 233 | 52 | Area_52 | Early_Auditory | 10 | 103 | 725, 580 |
| 54, 234 | A1 | Primary_Auditory_Cortex | Early_Auditory | 10 | 24 | 1023, 796 |
| 55, 235 | LBelt | Lateral_Belt_Complex | Early_Auditory | 10 | 174 | 820, 901 |
| 56, 236 | MBelt | Medial_Belt_Complex | Early_Auditory | 10 | 173 | 1242, 1236 |
| 57, 237 | PBelt | ParaBelt_Complex | Early_Auditory | 10 | 124 | 1719, 1439 |
| 58, 238 | PFcm | Area_PFcm | Early_Auditory | 10 | 105 | 1486, 1485 |
| 59, 239 | RI | RetroInsular_Cortex | Early_Auditory | 10 | 104 | 1149, 1334 |
| 60, 240 | A4 | Auditory_4_Complex | Auditory_Association | 11 | 175 | 3514, 3610 |
| 61, 241 | A5 | Auditory_5_Complex | Auditory_Association | 11 | 125 | 3346, 3881 |
| 62, 242 | STGa | Area_STGa | Auditory_Association | 11 | 123 | 2509, 2187 |
| 63, 243 | STSda | Area_STSd_anterior | Auditory_Association | 11 | 128 | 1944, 2389 |
| 64, 244 | STSdp | Area_STSd_posterior | Auditory_Association | 11 | 129 | 1994, 2605 |
| 65, 245 | STSva | Area_STSv_anterior | Auditory_Association | 11 | 176 | 1694, 1900 |
| 66, 246 | STSvp | Area_STSv_posterior | Auditory_Association | 11 | 130 | 2898, 2515 |
| 67, 247 | TA2 | Area_TA2 | Auditory_Association | 11 | 107 | 1518, 1726 |
| 68, 248 | AAIC | Anterior_Agranular_Insula_Complex | Insula_FrontalOperc | 12 | 112 | 1859, 1691 |
| 69, 249 | AVI | Anterior_Ventral_Insular_Area | Insula_FrontalOperc | 12 | 111 | 1446, 1792 |
| 70, 250 | FOP2 | Frontal_Opercular_Area_2 | Insula_FrontalOperc | 12 | 115 | 750, 720 |
| 71, 251 | FOP3 | Frontal_Opercular_Area_3 | Insula_FrontalOperc | 12 | 114 | 754, 614 |
| 72, 252 | FOP4 | Frontal_Opercular_Area_4 | Insula_FrontalOperc | 12 | 108 | 2522, 1678 |
| 73, 253 | FOP5 | Area_Frontal_Opercular_5 | Insula_FrontalOperc | 12 | 169 | 1297, 1365 |
| 74, 254 | Ig | Insular_Granular_Complex | Insula_FrontalOperc | 12 | 168 | 841, 1077 |
| 75, 255 | MI | Middle_Insular_Area | Insula_FrontalOperc | 12 | 109 | 2102, 1960 |
| 76, 256 | PI | Para-Insular_Area | Insula_FrontalOperc | 12 | 178 | 1033, 1058 |
| 77, 257 | Pir | Piriform_Cortex | Insula_FrontalOperc | 12 | 110 | 2287, 1856 |
| 78, 258 | PoI1 | Area_Posterior_Insular_1 | Insula_FrontalOperc | 12 | 167 | 1811, 1835 |
| 79, 259 | PoI2 | Posterior_Insular_Area_2 | Insula_FrontalOperc | 12 | 106 | 2747, 2675 |
| 80, 260 | H | Hippocampus | Medial_Temporal | 13 | 120 | 4283, 3626 |
| 81, 261 | PreS | PreSubiculum | Medial_Temporal | 13 | 119 | 1817, 1558 |
| 82, 262 | EC | Entorhinal_Cortex | Medial_Temporal | 13 | 118 | 2127, 2110 |
| 83, 263 | PeEc | Perirhinal_Ectorhinal_Cortex | Medial_Temporal | 13 | 122 | 4826, 4755 |
| 84, 264 | TF | Area_TF | Medial_Temporal | 13 | 135 | 3986, 4752 |
| 85, 265 | PHA1 | ParaHippocampal_Area_1 | Medial_Temporal | 13 | 126 | 1281, 1168 |
| 86, 266 | PHA2 | ParaHippocampal_Area_2 | Medial_Temporal | 13 | 155 | 783, 771 |
| 87, 267 | PHA3 | ParaHippocampal_Area_3 | Medial_Temporal | 13 | 127 | 2023, 1122 |
| 88, 268 | PHT | Area_PHT | Lateral_Temporal | 14 | 137 | 4182, 3410 |
| 89, 269 | TE1a | Area_TE1_anterior | Lateral_Temporal | 14 | 132 | 5227, 4180 |
| 90, 270 | TE1m | Area_TE1_Middle | Lateral_Temporal | 14 | 177 | 3339, 3429 |
| 91, 271 | TE1p | Area_TE1_posterior | Lateral_Temporal | 14 | 133 | 7116, 6010 |
| 92, 272 | TE2a | Area_TE2_anterior | Lateral_Temporal | 14 | 134 | 5691, 5753 |
| 93, 273 | TE2p | Area_TE2_posterior | Lateral_Temporal | 14 | 136 | 4115, 3040 |
| 94, 274 | TGd | Area_TG_dorsal | Lateral_Temporal | 14 | 131 | 10192, 10269 |
| 95, 275 | TGv | Area_TG_Ventral | Lateral_Temporal | 14 | 172 | 3694, 4515 |
| 96, 276 | PSL | PeriSylvian_Language_Area | TPO | 15 | 25 | 2154, 2759 |
| 97, 277 | STV | Superior_Temporal_Visual_Area | TPO | 15 | 28 | 2322, 2294 |
| 98, 278 | TPOJ1 | Area_TemporoParietoOccipital_Junction_1 | TPO | 15 | 139 | 2102, 3938 |
| 99, 279 | TPOJ2 | Area_TemporoParietoOccipital_Junction_2 | TPO | 15 | 140 | 1930, 2068 |
| 100, 280 | TPOJ3 | Area_TemporoParietoOccipital_Junction_3 | TPO | 15 | 141 | 1290, 1277 |
| 101, 281 | 7AL | Lateral_Area_7A | Superior_Parietal | 16 | 42 | 2134, 2030 |
| 102, 282 | 7Am | Medial_Area_7A | Superior_Parietal | 16 | 45 | 2995, 2379 |
| 103, 283 | 7PC | Area_7PC | Superior_Parietal | 16 | 47 | 3151, 3415 |
| 104, 284 | 7PL | Lateral_Area_7P | Superior_Parietal | 16 | 46 | 1695, 1363 |
| 105, 285 | 7Pm | Medial_Area_7P | Superior_Parietal | 16 | 29 | 1601, 1308 |
| 106, 286 | AIP | Anterior_IntraParietal_Area | Superior_Parietal | 16 | 117 | 1999, 2542 |
| 107, 287 | LIPd | Area_Lateral_IntraParietal_dorsal | Superior_Parietal | 16 | 95 | 1008, 869 |
| 108, 288 | LIPv | Area_Lateral_IntraParietal_ventral | Superior_Parietal | 16 | 48 | 1681, 1783 |
| 109, 289 | MIP | Medial_IntraParietal_Area | Superior_Parietal | 16 | 50 | 1872, 2403 |
| 110, 290 | VIP | Ventral_IntraParietal_Complex | Superior_Parietal | 16 | 49 | 1890, 1577 |
| 111, 291 | IP0 | Area_IntraParietal_0 | Inferior_Parietal | 17 | 146 | 1203, 1239 |
| 112, 292 | IP1 | Area_IntraParietal_1 | Inferior_Parietal | 17 | 145 | 1692, 1632 |
| 113, 293 | IP2 | Area_IntraParietal_2 | Inferior_Parietal | 17 | 144 | 2102, 1861 |
| 114, 294 | PF | Area_PF_Complex | Inferior_Parietal | 17 | 148 | 5457, 5251 |
| 115, 295 | PFm | Area_PFm_Complex | Inferior_Parietal | 17 | 149 | 8220, 8141 |
| 116, 296 | PFop | Area_PF_Opercular | Inferior_Parietal | 17 | 147 | 1797, 1783 |
| 117, 297 | PFt | Area_PFt | Inferior_Parietal | 17 | 116 | 1983, 2039 |
| 118, 298 | PGi | Area_PGi | Inferior_Parietal | 17 | 150 | 4791, 4970 |
| 119, 299 | PGp | Area_PGp | Inferior_Parietal | 17 | 143 | 2501, 3740 |
| 120, 300 | PGs | Area_PGs | Inferior_Parietal | 17 | 151 | 4552, 3366 |
| 121, 301 | 23d | Area_23d | Posterior_Cingulate | 18 | 32 | 1261, 1513 |
| 122, 302 | 31a | Area_31a | Posterior_Cingulate | 18 | 162 | 1260, 1116 |
| 123, 303 | 31pd | Area_31pd | Posterior_Cingulate | 18 | 161 | 1428, 864 |
| 124, 304 | 31pv | Area_31p_ventral | Posterior_Cingulate | 18 | 35 | 950, 1022 |
| 125, 305 | 7m | Area_7m | Posterior_Cingulate | 18 | 30 | 2128, 2067 |
| 126, 306 | d23ab | Area_dorsal_23_a+b | Posterior_Cingulate | 18 | 34 | 1607, 1106 |
| 127, 307 | DVT | Dorsal_Transitional_Visual_Area | Posterior_Cingulate | 18 | 142 | 1806, 2176 |
| 128, 308 | PCV | PreCuneus_Visual_Area | Posterior_Cingulate | 18 | 27 | 2245, 2416 |
| 129, 309 | POS1 | Parieto-Occipital_Sulcus_Area_1 | Posterior_Cingulate | 18 | 31 | 2531, 2727 |
| 130, 310 | POS2 | Parieto-Occipital_Sulcus_Area_2 | Posterior_Cingulate | 18 | 15 | 3261, 3093 |
| 131, 311 | ProS | ProStriate_Area | Posterior_Cingulate | 18 | 121 | 1222, 1055 |
| 132, 312 | RSC | RetroSplenial_Complex | Posterior_Cingulate | 18 | 14 | 2830, 3067 |
| 133, 313 | v23ab | Area_ventral_23_a+b | Posterior_Cingulate | 18 | 33 | 916, 1089 |
| 134, 314 | 10r | Area_10r | AntCing_MedPFC | 19 | 65 | 1589, 1053 |
| 135, 315 | 10v | Area_10v | AntCing_MedPFC | 19 | 88 | 3906, 2667 |
| 136, 316 | 25 | Area_25 | AntCing_MedPFC | 19 | 164 | 1911, 2135 |
| 137, 317 | 33pr | Area_33_prime | AntCing_MedPFC | 19 | 58 | 1354, 1316 |
| 138, 318 | 8BM | Area_8BM | AntCing_MedPFC | 19 | 63 | 3122, 3436 |
| 139, 319 | 9m | Area_9_Middle | AntCing_MedPFC | 19 | 69 | 6338, 5881 |
| 140, 320 | a24 | Area_a24 | AntCing_MedPFC | 19 | 61 | 2085, 2152 |
| 141, 321 | a24pr | Anterior_24_prime | AntCing_MedPFC | 19 | 59 | 1095, 1474 |
| 142, 322 | a32pr | Area_anterior_32_prime | AntCing_MedPFC | 19 | 179 | 1759, 1118 |
| 143, 323 | d32 | Area_dorsal_32 | AntCing_MedPFC | 19 | 62 | 2228, 2374 |
| 144, 324 | p24 | Area_posterior_24 | AntCing_MedPFC | 19 | 180 | 2394, 2442 |
| 145, 325 | p24pr | Area_Posterior_24_prime | AntCing_MedPFC | 19 | 57 | 1422, 1724 |
| 146, 326 | p32 | Area_p32 | AntCing_MedPFC | 19 | 64 | 1180, 1765 |
| 147, 327 | p32pr | Area_p32_prime | AntCing_MedPFC | 19 | 60 | 1569, 1305 |
| 148, 328 | pOFC | Posterior_OFC_Complex | AntCing_MedPFC | 19 | 166 | 2486, 2836 |
| 149, 329 | s32 | Area_s32 | AntCing_MedPFC | 19 | 165 | 604, 1015 |
| 150, 330 | 10d | Area_10d | OrbPolaFrontal | 20 | 72 | 3644, 3096 |
| 151, 331 | 10pp | Polar_10p | OrbPolaFrontal | 20 | 90 | 1997, 2487 |
| 152, 332 | 11l | Area_11l | OrbPolaFrontal | 20 | 91 | 3531, 3793 |
| 153, 333 | 13l | Area_13l | OrbPolaFrontal | 20 | 92 | 2429, 1757 |
| 154, 334 | 47m | Area_47m | OrbPolaFrontal | 20 | 66 | 799, 781 |
| 155, 335 | 47s | Area_47s | OrbPolaFrontal | 20 | 94 | 2795, 3080 |
| 156, 336 | a10p | Area_anterior_10p | OrbPolaFrontal | 20 | 89 | 1964, 1748 |
| 157, 337 | OFC | Orbital_Frontal_Complex | OrbPolaFrontal | 20 | 93 | 4560, 5232 |
| 158, 338 | p10p | Area_posterior_10p | OrbPolaFrontal | 20 | 170 | 2116, 2365 |
| 159, 339 | 44 | Area_44 | Inferior_Frontal | 21 | 74 | 2435, 2589 |
| 160, 340 | 45 | Area_45 | Inferior_Frontal | 21 | 75 | 3762, 2962 |
| 161, 341 | 47l | Area_47l_(47_lateral) | Inferior_Frontal | 21 | 76 | 2527, 2592 |
| 162, 342 | a47r | Area_anterior_47r | Inferior_Frontal | 21 | 77 | 4167, 3763 |
| 163, 343 | IFJa | Area_IFJa | Inferior_Frontal | 21 | 79 | 1513, 1405 |
| 164, 344 | IFJp | Area_IFJp | Inferior_Frontal | 21 | 80 | 960, 740 |
| 165, 345 | IFSa | Area_IFSa | Inferior_Frontal | 21 | 82 | 2057, 2641 |
| 166, 346 | IFSp | Area_IFSp | Inferior_Frontal | 21 | 81 | 1589, 1730 |
| 167, 347 | p47r | Area_posterior_47r | Inferior_Frontal | 21 | 171 | 2133, 1761 |
| 168, 348 | 46 | Area_46 | Dorsolateral_Prefrontal | 22 | 84 | 4863, 4394 |
| 169, 349 | 8Ad | Area_8Ad | Dorsolateral_Prefrontal | 22 | 68 | 3386, 3492 |
| 170, 350 | 8Av | Area_8Av | Dorsolateral_Prefrontal | 22 | 67 | 4807, 5902 |
| 171, 351 | 8BL | Area_8B_Lateral | Dorsolateral_Prefrontal | 22 | 70 | 3377, 4078 |
| 172, 352 | 8C | Area_8C | Dorsolateral_Prefrontal | 22 | 73 | 4085, 3134 |
| 173, 353 | 9-46d | Area_9-46d | Dorsolateral_Prefrontal | 22 | 86 | 4534, 4666 |
| 174, 354 | 9a | Area_9_anterior | Dorsolateral_Prefrontal | 22 | 87 | 3706, 3048 |
| 175, 355 | 9p | Area_9_Posterior | Dorsolateral_Prefrontal | 22 | 71 | 3426, 2488 |
| 176, 356 | a9-46v | Area_anterior_9-46v | Dorsolateral_Prefrontal | 22 | 85 | 3314, 2628 |
| 177, 357 | i6-8 | Inferior_6-8_Transitional_Area | Dorsolateral_Prefrontal | 22 | 97 | 1764, 2418 |
| 178, 358 | p9-46v | Area_posterior_9-46v | Dorsolateral_Prefrontal | 22 | 83 | 2871, 4635 |
| 179, 359 | s6-8 | Superior_6-8_Transitional_Area | Dorsolateral_Prefrontal | 22 | 98 | 1336, 2132 |
| 180, 360 | SFL | Superior_Frontal_Language_Area | Dorsolateral_Prefrontal | 22 | 26 | 3873, 3055 |

Column 1 (Reordered ID) shows the order in HCPex based on the HCP-MMP1_UniqueRegionList.csv, as described in the Methods, of the 360 cortical regions originally defined by Glasser et al (2016). The names of the cortical divisions shown in column 4 come from the same .csv file. The sixth column shows the original order used by Glasser et al (2016). Abbreviations: L=left hemisphere, R=right. MT+_Complex, MT+_Complex_and_Neighboring_Visual_Areas; SomaSens_Motor, Somatosensory_and_Motor; ParaCentral_MidCing, Paracentral_Lobular_and_Mid_Cingulate; Insula_FrontalOperc, Insular_and_Frontal_Opercular; TPO, Temporo-Parieto-Occipital_Junction; AntCing_MedPFC, Anterior_Cingulate_and_Medial_Prefrontal; OrbPolaFrontal, Orbital_and_Polar_Frontal.

**Neuroimaging.** The multi-modal imaging was collected using a standard Siemens Skyra 3T running VD13A SP4, with a standard Siemens 32-channel RF receive head coil. The resting-state functional brain imaging data used in this study were obtained and pre-processed by the UK Biobank. The details of the image acquisition are provided on the UK Biobank website in the form of a protocol (http://biobank.ctsu.ox.ac.uk/crystal/refer.cgi?id=2367). The UK Biobank conducted all the quality checking and data pre-processing procedures and the details of the pre-processing are available on the UK Biobank website (http://biobank.ctsu.ox.ac.uk/crystal/refer.cgi?id=1977) and elsewhere (Alfaro-Almagro, et al., 2018; Miller, et al., 2016). Briefly, data pre-processing was carried out using FSL (FMRIB Software Library) (Jenkinson, et al., 2012). All the data pre-processing procedures were performed by the UK Biobank team as described in (Miller, et al., 2016). The data pre-processing included correction for spatial and gradient distortions and head motion, intensity normalization and bias field removal, registration to the T1 weighted structural image, transformation to 2 mm Montreal Neurological Institute (MNI) space, and the FIX artefact removal procedure (Navarro Schröder, et al., 2015; Smith, et al., 2013). Finally, the head motion parameters were regressed out and structured artefacts were removed by ICA+FIX processing (Independent Component Analysis followed by FMRIB’s ICA-based X-noiseifier (Griffanti, et al., 2014; Salimi-Khorshidi, et al., 2014)). The data pre-processing pipeline developed by FMRIB (Oxford University Centre for Functional MRI of the Brain) and used in the pre-processing performed by the UK Biobank for the data used here has been widely used in resting-state fMRI studies (Colclough, et al., 2017; Navarro Schröder, et al., 2015; Smith, et al., 2015; Vidaurre, et al., 2018).

­
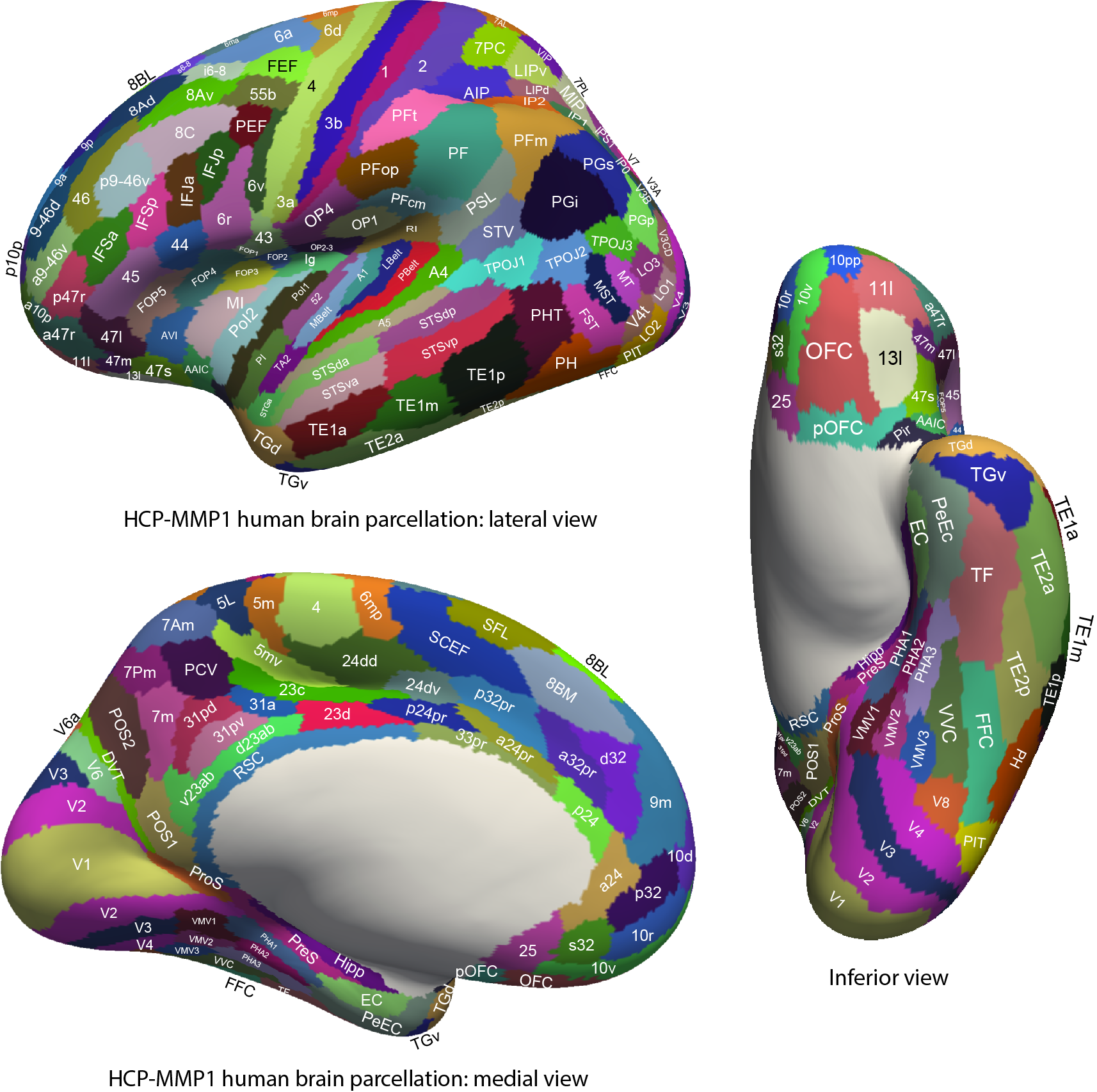


**Fig. S1. Anatomical regions of the human visual and other cortical regions.** Regions are shown as defined in the HCP-MMP atlas (Glasser, et al., 2016), and in its extended version HCPex (Huang, et al., 2022). The regions are shown on images of the human brain with the sulci expanded sufficiently to allow the regions within the sulci to be shown. Fig. S2 shows the brain without the sulci opened to help show which regions areas are normally visible. Abbreviations are provided in Table S1. (HCPBrainMaster4b.eps)


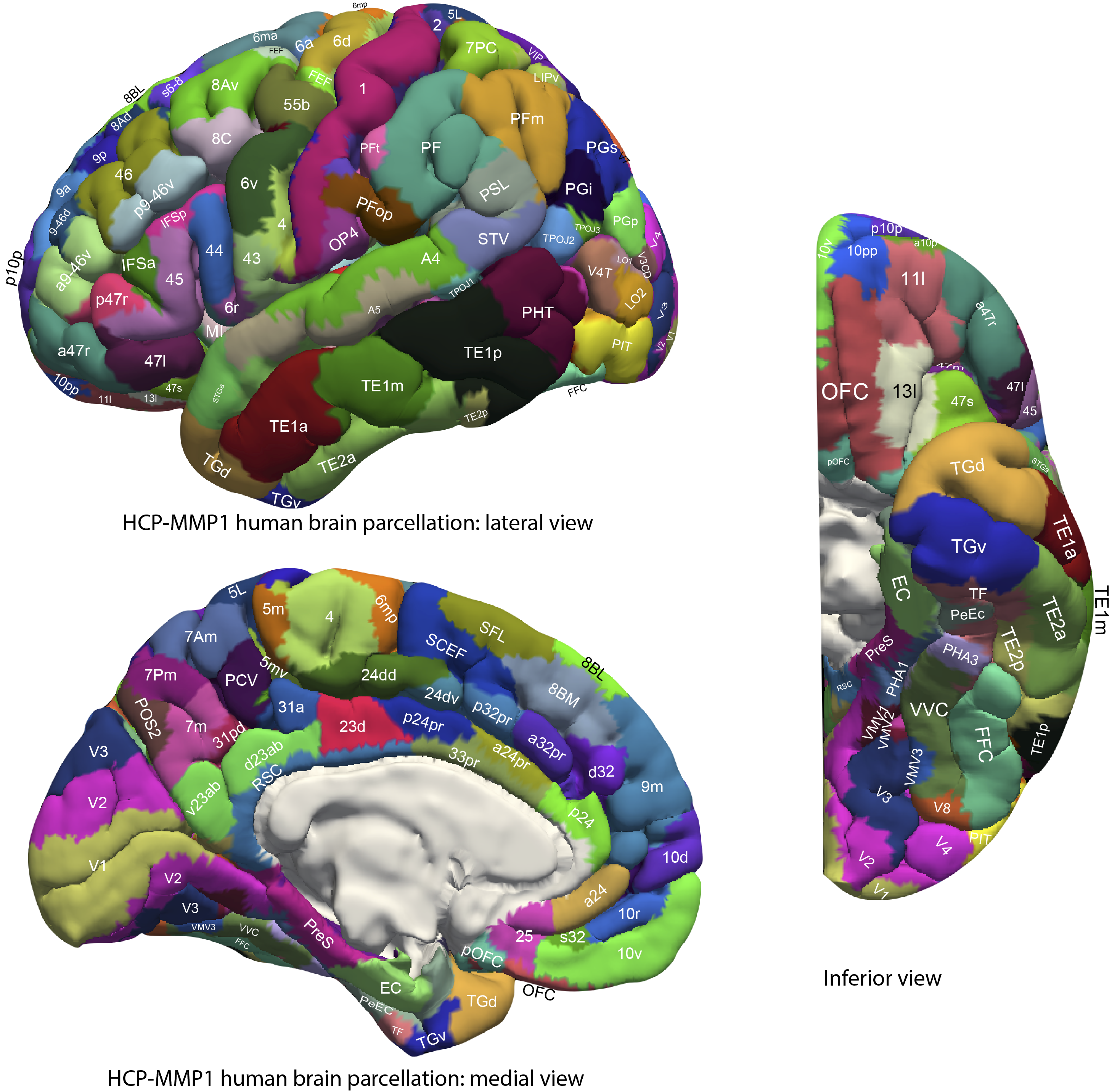


**Fig. S2. Anatomical regions of the human visual and other cortical regions**. Regions are shown as defined in the HCP-MMP atlas (Glasser, et al., 2016), and in its extended version HCPex (Huang, et al., 2022). The regions are shown on images of the human brain without the sulci expanded to show which cortical HCP-MMP regions are normally visible, for comparison with Fig. S1. (The ICBM153 MNI T1 image was used to prepare this figure.) Abbreviations are provided in Table S1. (HCPBrainMaster4c.eps)

**Table S2. The 50 top significant functional connectivity links in the HCPex atlas that were more negative in females**

| **Region 1** | **Region 2** | **t Value** | **Cohen's d** | **p Value** | **Region 1 Cortical Division** | **Region 2 Cortical Division** |
| --- | --- | --- | --- | --- | --- | --- |
| FOP4 L | 7m L | -54.97 | -0.58 | < 1.0e^-15^ | Insular and Frontal Opercular | Posterior Cingulate |
| MI L | 7m L | -51.55 | -0.54 | < 1.0e^-15^ | Insular and Frontal Opercular | Posterior Cingulate |
| 1 L | 24dd L | -50.99 | -0.53 | < 1.0e^-15^ | Somatosensory and Motor | Paracentral Lobular and Mid Cingulate |
| 3b L | 24dd L | -48.44 | -0.51 | < 1.0e^-15^ | Somatosensory and Motor | Paracentral Lobular and Mid Cingulate |
| FOP4 L | 31pd L | -48.37 | -0.51 | < 1.0e^-15^ | Insular and Frontal Opercular | Posterior Cingulate |
| 7m L | 9-46d L | -47.98 | -0.50 | < 1.0e^-15^ | Posterior Cingulate | Dorsolateral Prefrontal |
| FOP4 L | TE1m L | -47.92 | -0.50 | < 1.0e^-15^ | Insular and Frontal Opercular | Lateral Temporal |
| STSva L | FOP4 L | -46.46 | -0.49 | < 1.0e^-15^ | Auditory Association | Insular and Frontal Opercular |
| 7m L | 46 L | -45.44 | -0.48 | < 1.0e^-15^ | Posterior Cingulate | Dorsolateral Prefrontal |
| MI L | 31pd L | -45.20 | -0.47 | < 1.0e^-15^ | Insular and Frontal Opercular | Posterior Cingulate |
| 7m L | p32pr L | -45.14 | -0.47 | < 1.0e^-15^ | Posterior Cingulate | Anterior Cingulate and Medial Prefrontal |
| FOP4 L | 45 L | -45.05 | -0.47 | < 1.0e^-15^ | Insular and Frontal Opercular | Inferior Frontal |
| 24dv L | Ig L | -45.04 | -0.47 | < 1.0e^-15^ | Paracentral Lobular and Mid Cingulate | Insular and Frontal Opercular |
| FOP4 L | 31pv L | -44.95 | -0.47 | < 1.0e^-15^ | Insular and Frontal Opercular | Posterior Cingulate |
| 24dv L | FOP2 L | -44.94 | -0.47 | < 1.0e^-15^ | Paracentral Lobular and Mid Cingulate | Insular and Frontal Opercular |
| FOP2 L | p24pr L | -44.90 | -0.47 | < 1.0e^-15^ | Insular and Frontal Opercular | Anterior Cingulate and Medial Prefrontal |
| FOP4 L | POS1 L | -44.85 | -0.47 | < 1.0e^-15^ | Insular and Frontal Opercular | Posterior Cingulate |
| 23c L | FOP2 L | -44.77 | -0.47 | < 1.0e^-15^ | Paracentral Lobular and Mid Cingulate | Insular and Frontal Opercular |
| 24dv L | PreS L | -44.75 | -0.47 | < 1.0e^-15^ | Paracentral Lobular and Mid Cingulate | Medial Temporal |
| FOP4 L | PreS L | -44.62 | -0.47 | < 1.0e^-15^ | Insular and Frontal Opercular | Medial Temporal |
| STSvp L | FOP4 L | -44.61 | -0.47 | < 1.0e^-15^ | Auditory Association | Insular and Frontal Opercular |
| 43 L | OP1 L | -44.56 | -0.47 | < 1.0e^-15^ | Posterior Opercular | Posterior Opercular |
| PF L | 7m L | -44.55 | -0.47 | < 1.0e^-15^ | Inferior Parietal | Posterior Cingulate |
| PreS L | p24pr L | -44.34 | -0.46 | < 1.0e^-15^ | Medial Temporal | Anterior Cingulate and Medial Prefrontal |
| Ig L | p24pr L | -44.19 | -0.46 | < 1.0e^-15^ | Insular and Frontal Opercular | Anterior Cingulate and Medial Prefrontal |
| FOP4 L | PCV L | -44.16 | -0.46 | < 1.0e^-15^ | Insular and Frontal Opercular | Posterior Cingulate |
| 5mv L | 6v L | -44.10 | -0.46 | < 1.0e^-15^ | Paracentral Lobular and Mid Cingulate | Premotor |
| 23c L | PreS L | -44.07 | -0.46 | < 1.0e^-15^ | Paracentral Lobular and Mid Cingulate | Medial Temporal |
| 3a L | 24dv L | -44.01 | -0.46 | < 1.0e^-15^ | Somatosensory and Motor | Paracentral Lobular and Mid Cingulate |
| 5mv L | PreS L | -43.99 | -0.46 | < 1.0e^-15^ | Paracentral Lobular and Mid Cingulate | Medial Temporal |
| 24dd L | OP1 L | -43.99 | -0.46 | < 1.0e^-15^ | Paracentral Lobular and Mid Cingulate | Posterior Opercular |
| 24dd L | Ig L | -43.87 | -0.46 | < 1.0e^-15^ | Paracentral Lobular and Mid Cingulate | Insular and Frontal Opercular |
| 3b L | 24dv L | -43.75 | -0.46 | < 1.0e^-15^ | Somatosensory and Motor | Paracentral Lobular and Mid Cingulate |
| FOP1 L | PreS L | -43.73 | -0.46 | < 1.0e^-15^ | Posterior Opercular | Medial Temporal |
| 5mv L | FOP2 L | -43.71 | -0.46 | < 1.0e^-15^ | Paracentral Lobular and Mid Cingulate | Insular and Frontal Opercular |
| OP1 L | p24pr L | -43.70 | -0.46 | < 1.0e^-15^ | Posterior Opercular | Anterior Cingulate and Medial Prefrontal |
| FOP4 L | TGd L | -43.64 | -0.46 | < 1.0e^-15^ | Insular and Frontal Opercular | Lateral Temporal |
| 24dv L | 6v L | -43.63 | -0.46 | < 1.0e^-15^ | Paracentral Lobular and Mid Cingulate | Premotor |
| OP4 L | p24pr L | -43.58 | -0.46 | < 1.0e^-15^ | Posterior Opercular | Anterior Cingulate and Medial Prefrontal |
| A4 L | PreS L | -43.57 | -0.46 | < 1.0e^-15^ | Auditory Association | Medial Temporal |
| 24dv L | OP1 L | -43.53 | -0.46 | < 1.0e^-15^ | Paracentral Lobular and Mid Cingulate | Posterior Opercular |
| PGs L | p9-46v L | -43.49 | -0.46 | < 1.0e^-15^ | Inferior Parietal | Dorsolateral Prefrontal |
| FOP1 L | OP4 L | -43.42 | -0.46 | < 1.0e^-15^ | Posterior Opercular | Posterior Opercular |
| FOP4 L | POS2 L | -43.37 | -0.45 | < 1.0e^-15^ | Insular and Frontal Opercular | Posterior Cingulate |
| FOP4 L | 8Av L | -43.29 | -0.45 | < 1.0e^-15^ | Insular and Frontal Opercular | Dorsolateral Prefrontal |
| STSva L | MI L | -43.20 | -0.45 | < 1.0e^-15^ | Auditory Association | Insular and Frontal Opercular |
| 6v L | p24pr L | -42.97 | -0.45 | < 1.0e^-15^ | Premotor | Anterior Cingulate and Medial Prefrontal |
| 6r L | PGs L | -42.93 | -0.45 | < 1.0e^-15^ | Premotor | Inferior Parietal |
| 2 L | 24dd L | -42.89 | -0.45 | < 1.0e^-15^ | Somatosensory and Motor | Paracentral Lobular and Mid Cingulate |
| PFop L | 7m L | -42.87 | -0.45 | < 1.0e^-15^ | Inferior Parietal | Posterior Cingulate |

**Table S3. The 50 top significant functional connectivity links in the HCPex atlas that were more positive in females**

| **Region 1** | **Region 2** | **t Value** | **Cohen's d** | **p Value** | **Region 1 Cortical Division** | **Region 2 Cortical Division** |
| --- | --- | --- | --- | --- | --- | --- |
| 7m L | 10v L | 37.74 | 0.40 | < 1.0e^-15^ | Posterior Cingulate | Anterior Cingulate and Medial Prefrontal |
| 31pd L | 10v L | 29.04 | 0.30 | < 1.0e^-15^ | Posterior Cingulate | Anterior Cingulate and Medial Prefrontal |
| TE1a L | 7m L | 27.56 | 0.29 | < 1.0e^-15^ | Lateral Temporal | Posterior Cingulate |
| TE1m L | 10d L | 26.27 | 0.28 | < 1.0e^-15^ | Lateral Temporal | Orbital and Polar Frontal |
| POS2 L | 10v L | 26.12 | 0.27 | < 1.0e^-15^ | Posterior Cingulate | Anterior Cingulate and Medial Prefrontal |
| 31pv L | 10v L | 24.98 | 0.26 | < 1.0e^-15^ | Posterior Cingulate | Anterior Cingulate and Medial Prefrontal |
| TE1a L | POS2 L | 23.99 | 0.25 | < 1.0e^-15^ | Lateral Temporal | Posterior Cingulate |
| 31pd L | 7m L | 23.72 | 0.25 | < 1.0e^-15^ | Posterior Cingulate | Posterior Cingulate |
| 10d L | 8Ad L | 23.47 | 0.25 | < 1.0e^-15^ | Orbital and Polar Frontal | Dorsolateral Prefrontal |
| 10d L | p10p L | 23.02 | 0.24 | < 1.0e^-15^ | Orbital and Polar Frontal | Orbital and Polar Frontal |
| STSva L | 7m L | 23.01 | 0.24 | < 1.0e^-15^ | Auditory Association | Posterior Cingulate |
| POS1 L | 10v L | 22.80 | 0.24 | < 1.0e^-15^ | Posterior Cingulate | Anterior Cingulate and Medial Prefrontal |
| v23ab L | 10v L | 22.50 | 0.24 | < 1.0e^-15^ | Posterior Cingulate | Anterior Cingulate and Medial Prefrontal |
| PGs L | 10v L | 22.39 | 0.23 | < 1.0e^-15^ | Inferior Parietal | Anterior Cingulate and Medial Prefrontal |
| PFm L | 10d L | 21.40 | 0.22 | < 1.0e^-15^ | Inferior Parietal | Orbital and Polar Frontal |
| 7m L | 10r L | 21.39 | 0.22 | < 1.0e^-15^ | Posterior Cingulate | Anterior Cingulate and Medial Prefrontal |
| d23ab L | 10v L | 20.97 | 0.22 | < 1.0e^-15^ | Posterior Cingulate | Anterior Cingulate and Medial Prefrontal |
| 10d L | s6-8 L | 20.96 | 0.22 | < 1.0e^-15^ | Orbital and Polar Frontal | Dorsolateral Prefrontal |
| 10v L | 10pp L | 20.78 | 0.22 | < 1.0e^-15^ | Anterior Cingulate and Medial Prefrontal | Orbital and Polar Frontal |
| STSva L | PGs L | 20.24 | 0.21 | < 1.0e^-15^ | Auditory Association | Inferior Parietal |
| 10v L | 10d L | 20.24 | 0.21 | < 1.0e^-15^ | Anterior Cingulate and Medial Prefrontal | Orbital and Polar Frontal |
| 31a L | 10d L | 20.06 | 0.21 | < 1.0e^-15^ | Posterior Cingulate | Orbital and Polar Frontal |
| TE1m L | v23ab L | 19.96 | 0.21 | < 1.0e^-15^ | Lateral Temporal | Posterior Cingulate |
| PGs L | 10d L | 19.91 | 0.21 | < 1.0e^-15^ | Inferior Parietal | Orbital and Polar Frontal |
| 7m L | v23ab L | 19.53 | 0.20 | < 1.0e^-15^ | Posterior Cingulate | Posterior Cingulate |
| 10v L | 8Ad L | 19.45 | 0.20 | < 1.0e^-15^ | Anterior Cingulate and Medial Prefrontal | Dorsolateral Prefrontal |
| PGs L | 31pv L | 19.41 | 0.20 | < 1.0e^-15^ | Inferior Parietal | Posterior Cingulate |
| 10d L | 10pp L | 19.31 | 0.20 | < 1.0e^-15^ | Orbital and Polar Frontal | Orbital and Polar Frontal |
| PGi L | POS2 L | 19.23 | 0.20 | < 1.0e^-15^ | Inferior Parietal | Posterior Cingulate |
| 9m L | OFC L | 18.85 | 0.20 | < 1.0e^-15^ | Anterior Cingulate and Medial Prefrontal | Orbital and Polar Frontal |
| TE1a L | PFm L | 18.80 | 0.20 | < 1.0e^-15^ | Lateral Temporal | Inferior Parietal |
| 7m L | 10d L | 18.79 | 0.20 | < 1.0e^-15^ | Posterior Cingulate | Orbital and Polar Frontal |
| TE1m L | 7m L | 18.74 | 0.20 | < 1.0e^-15^ | Lateral Temporal | Posterior Cingulate |
| PGs L | 10r L | 18.48 | 0.19 | < 1.0e^-15^ | Inferior Parietal | Anterior Cingulate and Medial Prefrontal |
| 7Pm L | 10v L | 17.90 | 0.19 | < 1.0e^-15^ | Superior Parietal | Anterior Cingulate and Medial Prefrontal |
| 31pv L | 7m L | 17.75 | 0.19 | < 1.0e^-15^ | Posterior Cingulate | Posterior Cingulate |
| STSva L | 10v L | 17.63 | 0.18 | < 1.0e^-15^ | Auditory Association | Anterior Cingulate and Medial Prefrontal |
| PGs L | POS1 L | 17.32 | 0.18 | < 1.0e^-15^ | Inferior Parietal | Posterior Cingulate |
| 31a L | 10v L | 17.09 | 0.18 | < 1.0e^-15^ | Posterior Cingulate | Anterior Cingulate and Medial Prefrontal |
| d23ab L | 10pp L | 17.04 | 0.18 | < 1.0e^-15^ | Posterior Cingulate | Orbital and Polar Frontal |
| OFC L | 8BL L | 16.97 | 0.18 | < 1.0e^-15^ | Orbital and Polar Frontal | Dorsolateral Prefrontal |
| 7m L | 9m L | 16.72 | 0.18 | < 1.0e^-15^ | Posterior Cingulate | Anterior Cingulate and Medial Prefrontal |
| 31pv L | 10pp L | 16.69 | 0.18 | < 1.0e^-15^ | Posterior Cingulate | Orbital and Polar Frontal |
| TE1m L | 10v L | 16.68 | 0.17 | < 1.0e^-15^ | Lateral Temporal | Anterior Cingulate and Medial Prefrontal |
| TE1a L | 7Pm L | 16.29 | 0.17 | < 1.0e^-15^ | Lateral Temporal | Superior Parietal |
| 10v L | p10p L | 16.24 | 0.17 | < 1.0e^-15^ | Anterior Cingulate and Medial Prefrontal | Orbital and Polar Frontal |
| STSva L | TE1m L | 16.20 | 0.17 | < 1.0e^-15^ | Auditory Association | Lateral Temporal |
| TE1a L | PGs L | 16.18 | 0.17 | < 1.0e^-15^ | Lateral Temporal | Inferior Parietal |
| TE1m L | 31pd L | 16.10 | 0.17 | < 1.0e^-15^ | Lateral Temporal | Posterior Cingulate |
| PCV L | 10v L | 15.97 | 0.17 | < 1.0e^-15^ | Posterior Cingulate | Anterior Cingulate and Medial Prefrontal |

**Analysis of sex differences in Functional Connectivity**

The functional connectivity differences between females (N=19,396) and males (N=17,135) are shown in Fig. 3 in terms of effect size measured by Cohen’s d. The connectivity differences between females and males were similar for the right hemisphere, as shown in Fig. S3.

Fig. 5 in the main text shows the differences of functional connectivity for females – males with the standard covariates regressed out, together with Field 6032 **maximum workload during fitness test** and Field 23100 **whole body fat mass,** in the two-sample t-tests. The results were from 2099 females and 1889 males. In Fig. S4 we show for the identical participants the same analysis but with only the standard covariates regressed out. (The standard covariates regressed out were Age, BMI, Education qualification, smoking status, drinker status, Townsend deprivation index, head motion, and imaging site information.) Comparison of Fig. S4 with Fig. 5 shows that regressing out these two additional covariates did reduce greatly the differences of functional connectivities for females – males, when exactly the same participants are included. Some measures of these effects are provided in Table 2. The mean Cohen’s d for the difference of FC for females – males reduced from -0.18 to -0.06 when these two covariates are added, with total body fat mass being more involved than the workload measure (Table 2). A paired t-test showed that the Cohen’s d matrix in Fig. 5 was significantly greater than that of Fig. S4 (t = 254.0, p < 1.0e^-15^). This indicated that the differences of FC for females – males were greatly reduced by regressing out these two covariates.


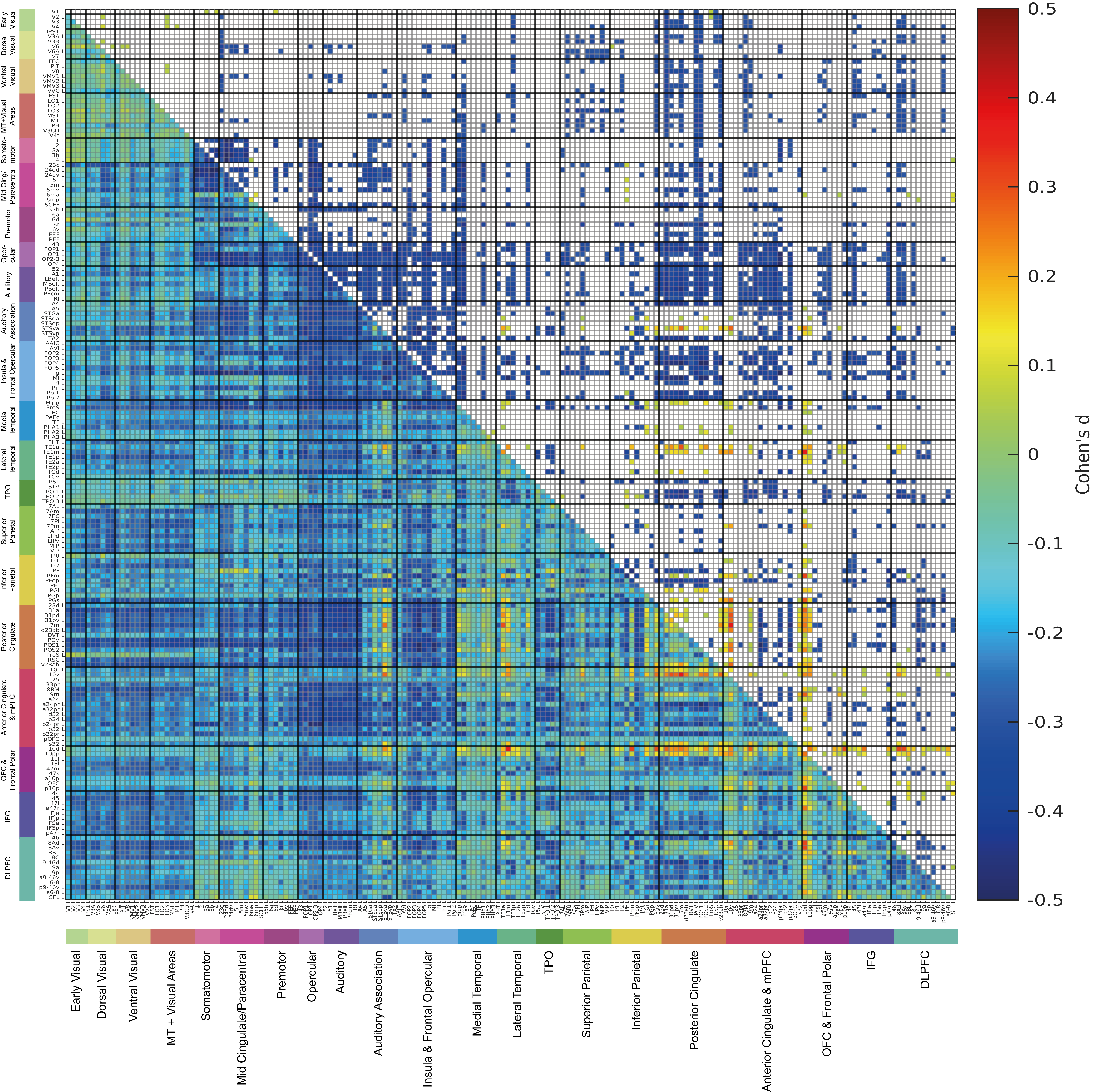


Fig. S3. The lower left triangle shows the matrix of functional connectivity differences for females – males with the Cohen’s d values showing the effect size of the differences. The matrix is for the functional connectivities in the right hemisphere, as listed in Table S1, with V1, V2, V3 … at the top of the y axis and the left of the x axis. The upper triangle matrix shows the Cohen’s d values of top 20% significant negative links and all significant positive links after Bonferroni correction (corrected p < 3.1e^-6^). These results were from 19396 females and 17135 males. The negative values shown in the upper right triangle had d < -0.28, and all the values shown in the matrix were in the range from -0.5 to 0.5. The standard covariates regressed out in this analysis were Age, BMI, Education qualification, smoking status, drinker status, Townsend deprivation index, head motion, and imaging site information.

(UKBdata_d_matrix_Female_vs_Male_BonCorrection_top20neg_pos_RH_22Aug.eps)


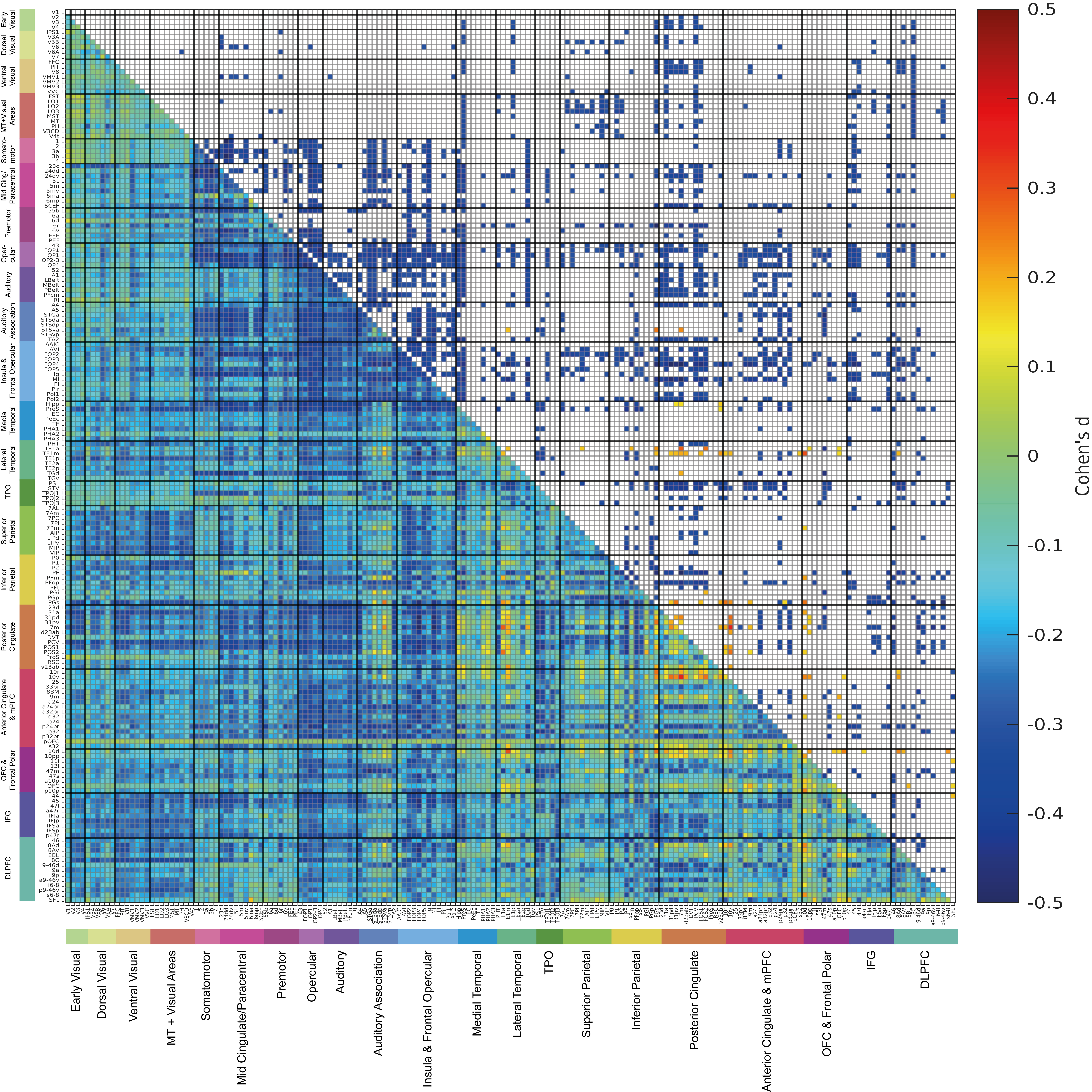


Fig. S4. The lower left triangle shows the matrix of functional connectivity differences for females - males with the Cohen’s d values showing the effect size of the differences. The matrix is for the functional connectivities in the left hemisphere, as listed in Table S1, with V1, V2, V3 … at the top of the y axis and the left of the x axis. The upper triangle matrix shows the Cohen’s d values of top 20% significant negative links and all significant positive links after Bonferroni correction (corrected p < 3.1e^-6^). These results were from the same 2099 females and 1889 males included in Fig. 5, which had two more covariates regressed out. Fig. S4 has only the standard covariates regressed out. All the values shown in the matrix are in the range from -0.5 to 0.5. The standard covariates regressed out in this analysis were Age, BMI, Education qualification, smoking status, drinker status, Townsend deprivation index, head motion, and imaging site information.

(UKBdata_d_matrix_Female_vs_Male_BonCorrection_top20neg_pos_less_subjects_22Aug.eps)

**Links that were higher in females than males in the UK Biobank dataset**

To analyse the functional implications of the functional connectivity links that were higher in females, correlations between these functional connectivity links and the behavioural measures available in the UK Biobank dataset were analysed. Key findings are presented next.

Fig. S5 shows that among the 356 links that were greater in females (indicated by non-white regions in Fig. S5), links that were significantly correlated with happiness and well-being included links in the medial orbitofrontal cortex (OFC), and ventromedial prefrontal cortex (10v, 10r and s32), all of which are reward regions implicated in emotion (Rolls, et al., 2023a).


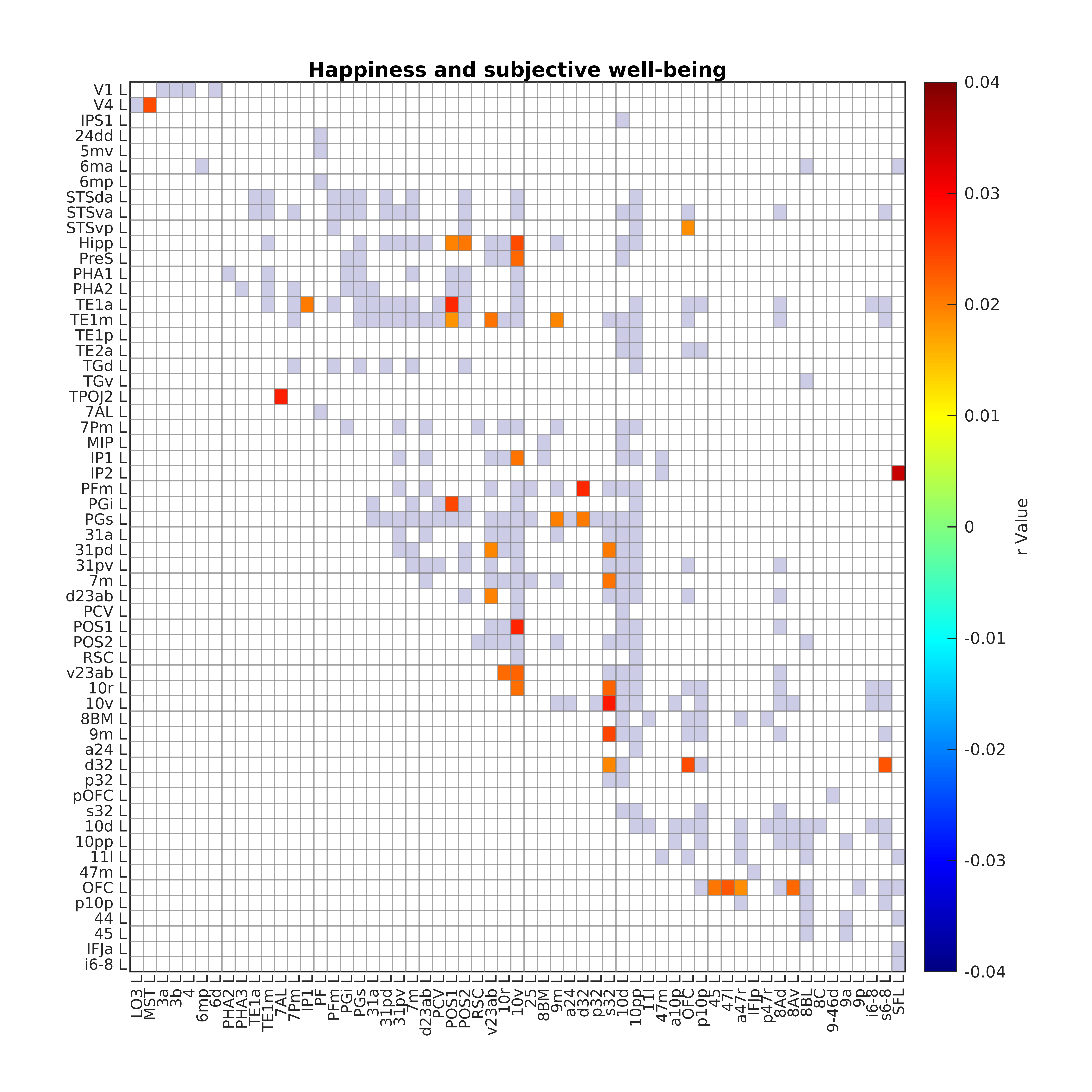


Fig. S5. The correlation (r) matrix shows the Functional Connectivities significantly correlated with the scores of the UK Biobank category ‘Happiness and subjective well-being’ (FDR-corrected p < 0.05). All the values shown in the matrix were in the range from -0.04 to 0.04 to enable comparison with Fig. S6. A total of 358 links in this matrix that are not white are those that were significantly higher in females. The standard covariates regressed out in this analysis were Age, BMI, Education qualification, smoking status, drinker status, Townsend deprivation index, head motion, and imaging site information. These results were from 23,958 participants.

Fig. S6 shows that some of the links higher in females were correlated with performance on the Tower rearranging task in the UK Biobank, which is a cognitive rearrangement task with a considerable memory load. These links included the posterior cingulate cortex (v23, POS2) with in some cases the hippocampal memory system (Hipp, Presubiculum, PHA1 and PHA2), both of which are implicated in memory (Rolls, et al., 2022; Rolls, et al., 2023b).


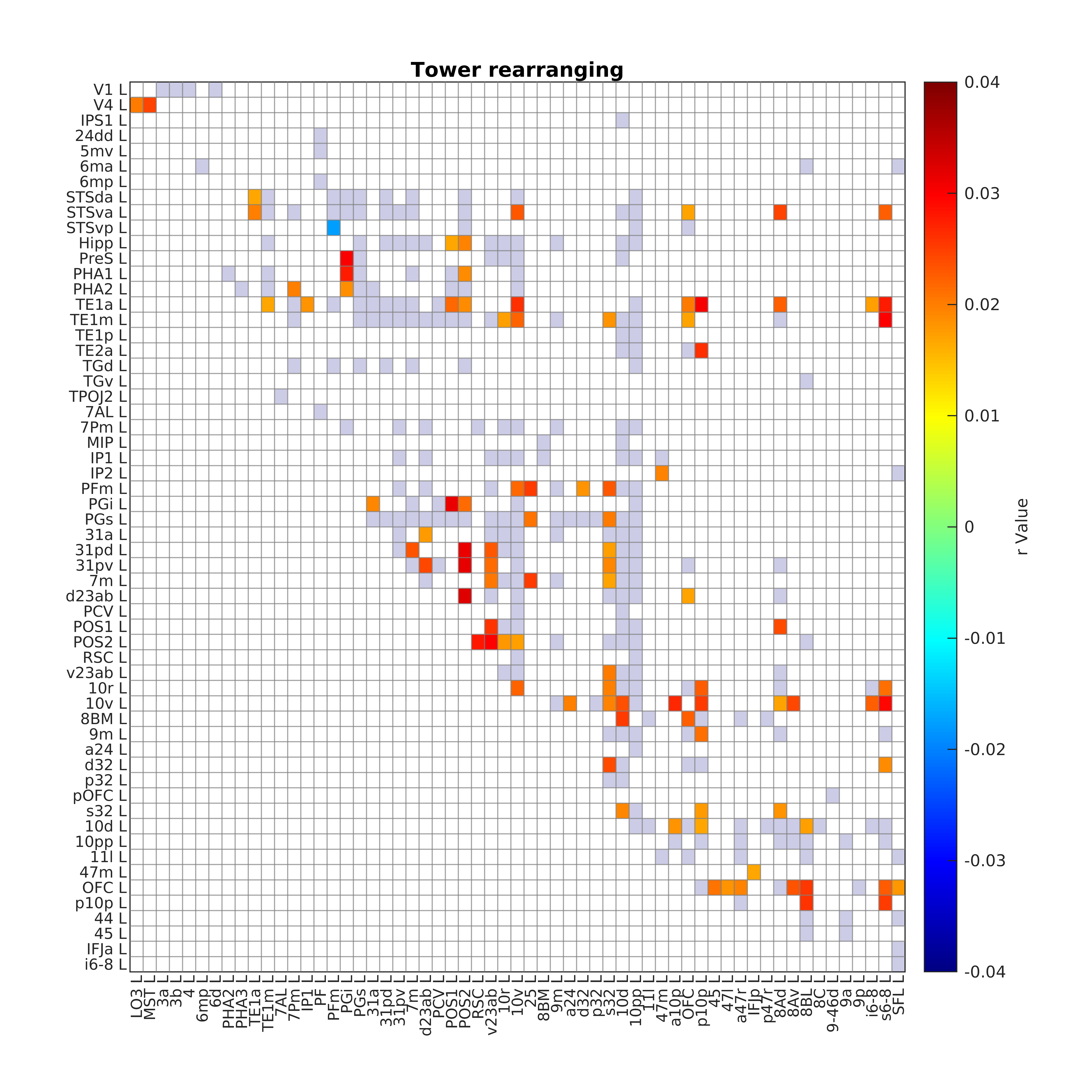


Fig. S6. The correlation (r) matrix shows the Functional Connectivities significantly correlated with the scores of the UK Biobank Field 21004 from ‘Tower rearranging’ (FDR-corrected p < 0.05). All the values shown in the matrix were in the range from -0.04 to 0.04 to enable comparison with Fig. S5. A total of 358 links in this matrix that are not white are those that were significantly higher in females. The standard covariates regressed out in this analysis were Age, BMI, Education qualification, smoking status, drinker status, Townsend deprivation index, head motion, and imaging site information. These results were from 22,059 participants.


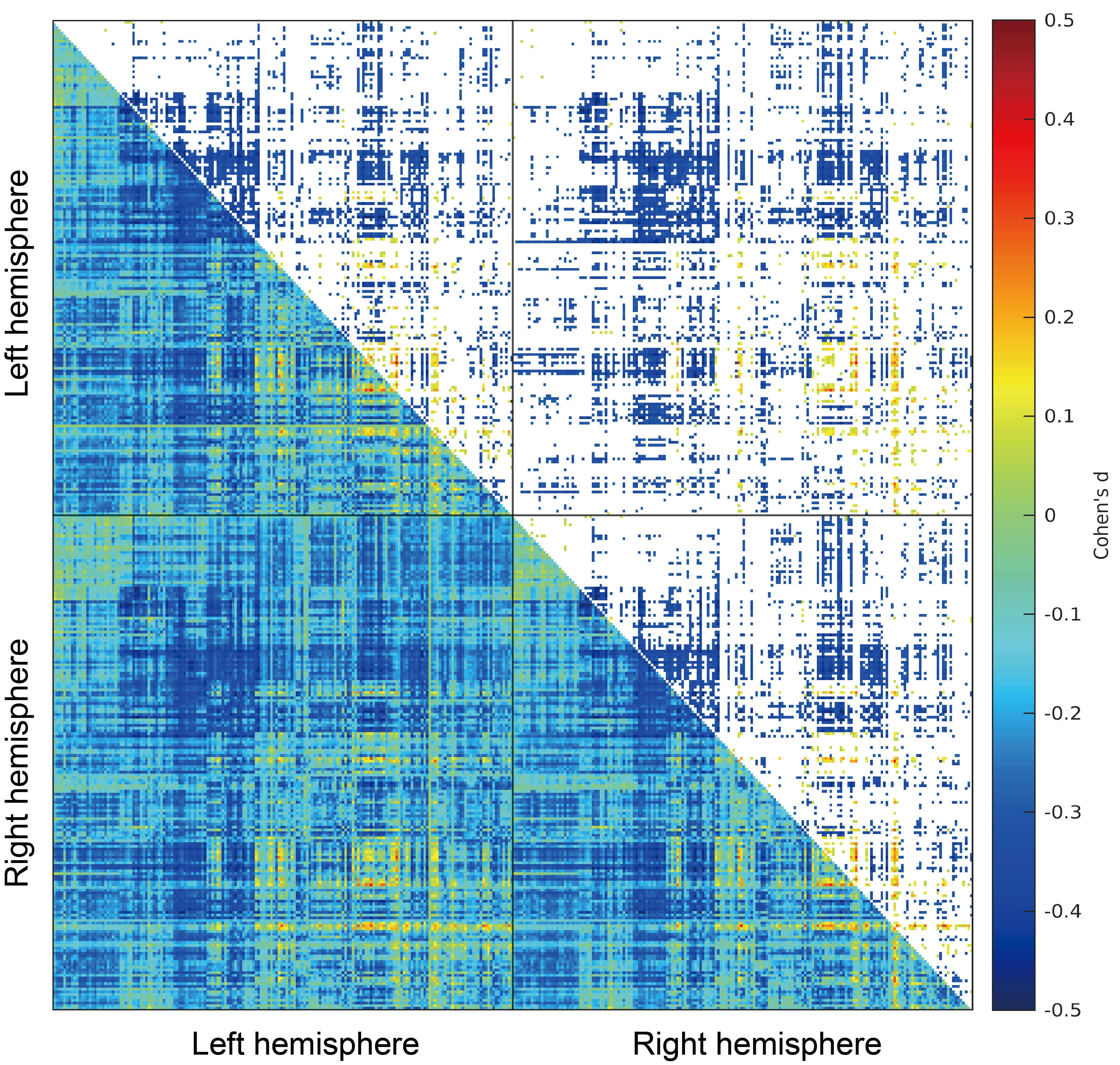


Fig. S7. The Cohen’s d values for the differences of functional connectivity for females – males for all 360 cortical regions in the HCP-MMP atlas, with the same order of regions and the same conventions as in Fig. 3. The upper right quadrant shows the functional connectivities between the Left and the Right hemisphere. Interestingly, the functional connectivity differences between females and males within each hemisphere were also in general found also for the connectivities between the two hemispheres. The standard covariates regressed out in this analysis were Age, BMI, education qualifications, smoking status, drinker status, Townsend deprivation index, head motion, and imaging site information. The cortical regions in the HCP-MMP atlas are illustrated in Figs. S1 and S2, and their names and divisions are shown in Table S1.

References

Alfaro-Almagro, F., Jenkinson, M., Bangerter, N.K., Andersson, J.L.R., Griffanti, L., Douaud, G., Sotiropoulos, S.N., Jbabdi, S., Hernandez-Fernandez, M., Vallee, E., Vidaurre, D., Webster, M., McCarthy, P., Rorden, C., Daducci, A., Alexander, D.C., Zhang, H., Dragonu, I., Matthews, P.M., Miller, K.L., Smith, S.M. (2018) Image processing and Quality Control for the first 10,000 brain imaging datasets from UK Biobank. Neuroimage, 166:400-424.

Colclough, G.L., Smith, S.M., Nichols, T.E., Winkler, A.M., Sotiropoulos, S.N., Glasser, M.F., Van Essen, D.C., Woolrich, M.W. (2017) The heritability of multi-modal connectivity in human brain activity. Elife, 6.

Glasser, M.F., Coalson, T.S., Robinson, E.C., Hacker, C.D., Harwell, J., Yacoub, E., Ugurbil, K., Andersson, J., Beckmann, C.F., Jenkinson, M., Smith, S.M., Van Essen, D.C. (2016) A multi-modal parcellation of human cerebral cortex. Nature, 536:171-8.

Griffanti, L., Salimi-Khorshidi, G., Beckmann, C.F., Auerbach, E.J., Douaud, G., Sexton, C.E., Zsoldos, E., Ebmeier, K.P., Filippini, N., Mackay, C.E., Moeller, S., Xu, J., Yacoub, E., Baselli, G., Ugurbil, K., Miller, K.L., Smith, S.M. (2014) ICA-based artefact removal and accelerated fMRI acquisition for improved resting state network imaging. Neuroimage, 95:232-47.

Huang, C.-C., Rolls, E.T., Feng, J., Lin, C.-P. (2022) An extended Human Connectome Project multimodal parcellation atlas of the human cortex and subcortical areas. Brain Structure and Function, 227:763-778.

Jenkinson, M., Beckmann, C.F., Behrens, T.E., Woolrich, M.W., Smith, S.M. (2012) Fsl. Neuroimage, 62:782-790.

Miller, K.L., Alfaro-Almagro, F., Bangerter, N.K., Thomas, D.L., Yacoub, E., Xu, J., Bartsch, A.J., Jbabdi, S., Sotiropoulos, S.N., Andersson, J.L. (2016) Multimodal population brain imaging in the UK Biobank prospective epidemiological study. Nature neuroscience, 19:1523-1536.

Navarro Schröder, T., Haak, K.V., Zaragoza Jimenez, N.I., Beckmann, C.F., Doeller, C.F. (2015) Functional topography of the human entorhinal cortex. Elife, 4.

Rolls, E.T., Deco, G., Huang, C.C., Feng, J. (2022) The effective connectivity of the human hippocampal memory system. Cereb. Cortex, 32:3706-3725.

Rolls, E.T., Deco, G., Huang, C.C., Feng, J. (2023a) The human orbitofrontal cortex, vmPFC, and anterior cingulate cortex effective connectome: emotion, memory, and action. Cereb. Cortex, 33:330-359.

Rolls, E.T., Wirth, S., Deco, G., Huang, C.-C., Feng, J. (2023b) The human posterior cingulate, retrosplenial and medial parietal cortex effective connectome, and implications for memory and navigation. Hum. Brain Mapp., 44:629-655.

Salimi-Khorshidi, G., Douaud, G., Beckmann, C.F., Glasser, M.F., Griffanti, L., Smith, S.M. (2014) Automatic denoising of functional MRI data: combining independent component analysis and hierarchical fusion of classifiers. Neuroimage, 90:449-68.

Smith, S.M., Beckmann, C.F., Andersson, J., Auerbach, E.J., Bijsterbosch, J., Douaud, G., Duff, E., Feinberg, D.A., Griffanti, L., Harms, M.P., Kelly, M., Laumann, T., Miller, K.L., Moeller, S., Petersen, S., Power, J., Salimi-Khorshidi, G., Snyder, A.Z., Vu, A.T., Woolrich, M.W., Xu, J., Yacoub, E., Ugurbil, K., Van Essen, D.C., Glasser, M.F., Consortium, W.U.-M.H. (2013) Resting-state fMRI in the Human Connectome Project. Neuroimage, 80:144-68.

Smith, S.M., Nichols, T.E., Vidaurre, D., Winkler, A.M., Behrens, T.E., Glasser, M.F., Ugurbil, K., Barch, D.M., Van Essen, D.C., Miller, K.L. (2015) A positive-negative mode of population covariation links brain connectivity, demographics and behavior. Nat. Neurosci., 18:1565-7.

Vidaurre, D., Abeysuriya, R., Becker, R., Quinn, A.J., Alfaro-Almagro, F., Smith, S.M., Woolrich, M.W. (2018) Discovering dynamic brain networks from big data in rest and task. Neuroimage, 180:646-656.
